# α-parvin controls chondrocyte column formation and regulates long bone development

**DOI:** 10.1038/s41413-023-00284-7

**Published:** 2023-08-22

**Authors:** Jifan Yuan, Ling Guo, Jiaxin Wang, Zhongjun Zhou, Chuanyue Wu

**Affiliations:** 1 Guangdong Provincial Key Laboratory of Cell Microenvironment and Disease Research, Shenzhen Key Laboratory of Cell Microenvironment, Department of Biology, School of Life Sciences, Southern University of Science and Technology, Shenzhen, 518055 China; 2 School of Biomedical Sciences, Li Ka Shing Faculty of Medicine, The University of Hong Kong, Hong Kong, 999077 China; 3 Shenzhen Key Laboratory of Epigenetics and Precision Medicine for Cancers, National Cancer Center/National Clinical Research Center for Cancer/Cancer Hospital & Shenzhen Hospital, Chinese Academy of Medical Sciences and Peking Union Medical College, Shenzhen, China; 4 Department of Pathology, School of Medicine, University of Pittsburgh, Pittsburgh, PA 15261 USA

**Keywords:** Bone, Bone quality and biomechanics

## Abstract

Endochondral ossification requires proper control of chondrocyte proliferation, differentiation, survival, and organization. Here we show that knockout of α-parvin, an integrin-associated focal adhesion protein, from murine limbs causes defects in endochondral ossification and dwarfism. The mutant long bones were shorter but wider, and the growth plates became disorganized, especially in the proliferative zone. With two-photon time-lapse imaging of bone explant culture, we provide direct evidence showing that α-parvin regulates chondrocyte rotation, a process essential for chondrocytes to form columnar structure. Furthermore, loss of α-parvin increased binucleation, elevated cell death, and caused dilation of the resting zones of mature growth plates. Single-cell RNA-seq analyses revealed alterations of transcriptome in all three zones (i.e., resting, proliferative, and hypertrophic zones) of the growth plates. Our results demonstrate a crucial role of α-parvin in long bone development and shed light on the cellular mechanism through which α-parvin regulates the longitudinal growth of long bones.

## Introduction

Vertebrate long bone development is driven by growth plate, a structure that is composed of multiple layers of chondrocytes surrounded by dense cartilage extracellular matrix (ECM). Nutrients, gas, and growth factors diffuse into the growth plate and modulate the growth of long bone.^1^ Chondrocytes within the growth plate are divided into three layers: resting zone, proliferative zone, and hypertrophic zone. Resting zone harbors round shape chondrocytes capable of generating proliferative columns in neonatal or fetal mice. Resting zone chondrocytes acquire self-renewal capacity and become long-term chondroprogenitors in mature growth plates.^2–4^ In the proliferative zone, chondrocytes become flattened in shape. They divide, rotate, and finally stack into columnar structures.^5^ The hypertrophic zone localizes below the proliferative zone. Chondrocytes from the upper layer gradually enlarge within the hypertrophic zone and secret type X collagen rather than type II collagen. They are fated to cell death or trans-differentiation at the chondro-osseous junction.^6,7^ Proliferation and hypertrophy of growth plate chondrocytes result in longitudinal displacement and provide a cartilaginous template for subsequent ossification, thus elongating the long bones.^8,9^

Integrin adhesion complexes control cell-ECM adhesion and various biological events by transmitting signals from the ECM.^10–12^ Previous studies have underscored the importance of integrins in skeletal homeostasis and development from aspects of either cartilage or bone in the past few decades.^13–15^ Integrin β1 is a major isoform of integrin expressed by chondrocytes.^16^ Previous studies have shown that chondrocyte-specific deletion of integrin β1 using Col2a1-Cre or Prx1-Cre caused severe chondrodysplasia and dwarfism in mice.^13,17^ However, how integrin signaling regulates bone development is incompletely understood.

α-parvin is a widely expressed and evolutionally conserved component of integrin adhesion complexes. α-parvin forms a ternary protein complex with PINCH and integrin-linked kinase (ILK) and anchors the integrins to the actin cytoskeleton and signaling.^18,19^ Formation of the Pinch-ILK-Parvin complex prevents the degradation of the components of this protein complex.^20,21^ Disruption of the PINCH-ILK-Parvin complex in various tissue and organisms results in severe defects and embryonic lethality.^22^ Previous studies have revealed important roles of α-parvin in several cell types and organ systems. For example, constitutive ablation of α-parvin resulted in embryonic lethality with severe defects in the cardiovascular system and renal agenesis.^23,24^ Endothelial α-parvin regulates vascular integrity and cell-cell junction maintenance.^25,26^ Epidermis-specific deletion of α-parvin resulted in progressive alopecia. α-parvin deficient keratinocytes showed adhesion defect, impaired polarity, and abnormal differentiation.^27^ However, the functions of α-parvin in chondrocytes and long bone development were largely unknown.

Given the intimate interaction between chondrocytes and the surrounding ECM, we hypothesized that α-parvin plays an important role in regulation of chondrocyte behavior and long bone development. In this study, we have tested this hypothesis by conditionally knocking out α-parvin in the limb buds of mice. Furthermore, we have employed a combination of molecular, cellular, and two-photon live imaging to investigate the functions of α-parvin in chondrocytes and long bone development. Our results demonstrate important roles of α-parvin in chondrocyte post-division rotation, column formation, and long bone development. We describe below our findings in detail.

## Results

### Limb bud deletion of α-parvin results in short stature in mice

To investigate the functions of α-parvin in long bone development, we generated *PARVA*^*flox/flox*^ *Prx1*^*cre*^ mice (cKO mice), in which α-parvin was ablated from the limb bud mesoderm. The cKO mice, irrespective of male or female, died before two months of age. In addition, cKO mice were produced at a ratio lower than that Mendelian law predicted, possibly due to localization of the genes encoding α-parvin and Prx1-Cre in the same chromosome, as both genes are known to localize in chromosome 7.^28^

**Fig. 1:**
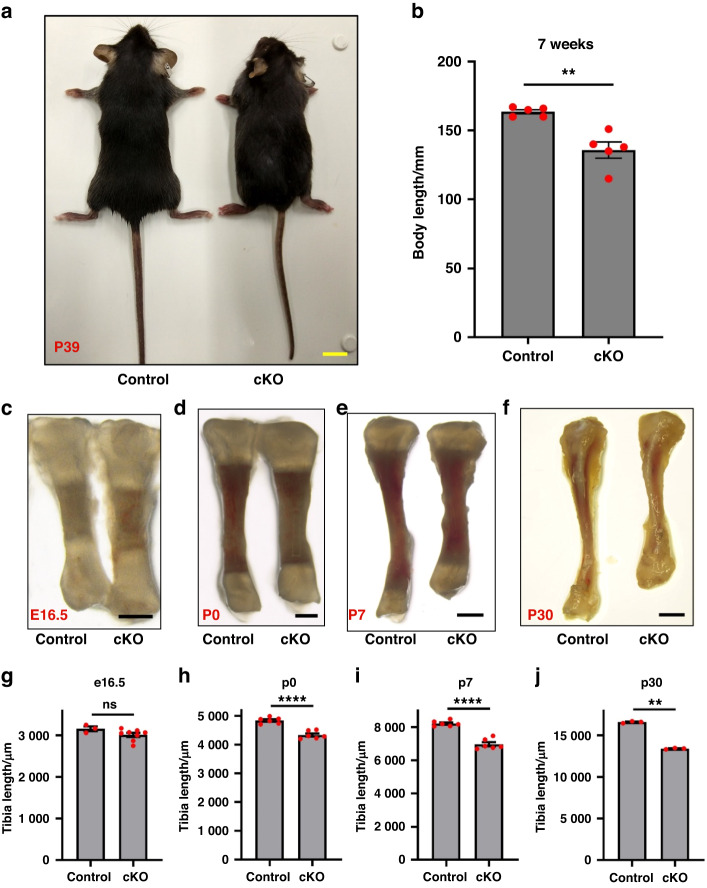
Loss of α-Parvin leads to dwarfism and shorter long bones in mice. **a** Gross morphology of six weeks-old control and *PARVA*^*flox/flox*^ *Prx1*^*cre*^ mice (cKO mice). Scale bar = 1 cm. **b** Comparison of body length between control and cKO mice at 7 weeks of age. **P < 0.01. paired t-test. mean ± s.e.m. *n* = 5 mice for each group. **c**–**f** Stereoscopic images of tibia from E16.5, P0, P7, and P30 control (left) and cKO mice (right). Scale bars = 500 μm, 500 μm, 1 mm, 2 mm respectively (from left to right). **g** Comparison of tibia length between control and cKO embryos at E16.5. ns not significant. unpaired t-test. mean ± s.e.m. *n* = 3 control and 8 cKO embryos from the same litter. **h** Comparison of tibia length between control and cKO mice at P0. ****P < 0.000 1. paired t-test. mean ± s.e.m. *n* = 6 mice for each group. **i** Comparison of tibia length between control and cKO mice at P7. ****P < 0.000 1. paired t-test. mean ± s.e.m. *n* = 6 mice for each group. **j** Comparison of tibia length between control and cKO mice at P30. **P < 0.01. paired t-test. mean ± s.e.m. *n* = 3 mice for each group

The cKO mice showed significant dwarfism starting from the adolescent stage (Fig. 1a). They were 15% shorter in body length at seven weeks of age compared to control mice (Fig. 1b). As for the long bone development, the tibia of cKO mice was similar in length to control at E16.5 (Fig. 1c, g) but became ~10% shorter than that of control mice at P0 (postnatal day 0) (Fig. 1d, h). The difference in tibia length became more evident in elder cKO and control mice. P7 and P30 cKO mice all showed a 15% to 20% reduction in tibia length compared to control mice (Fig. 1e, f, i, j). These results suggest that α-parvin is required for proper long bone growth, especially at the postnatal stage.

### Limb bud deletion of α-parvin causes widening of long bones

**Fig. 2:**
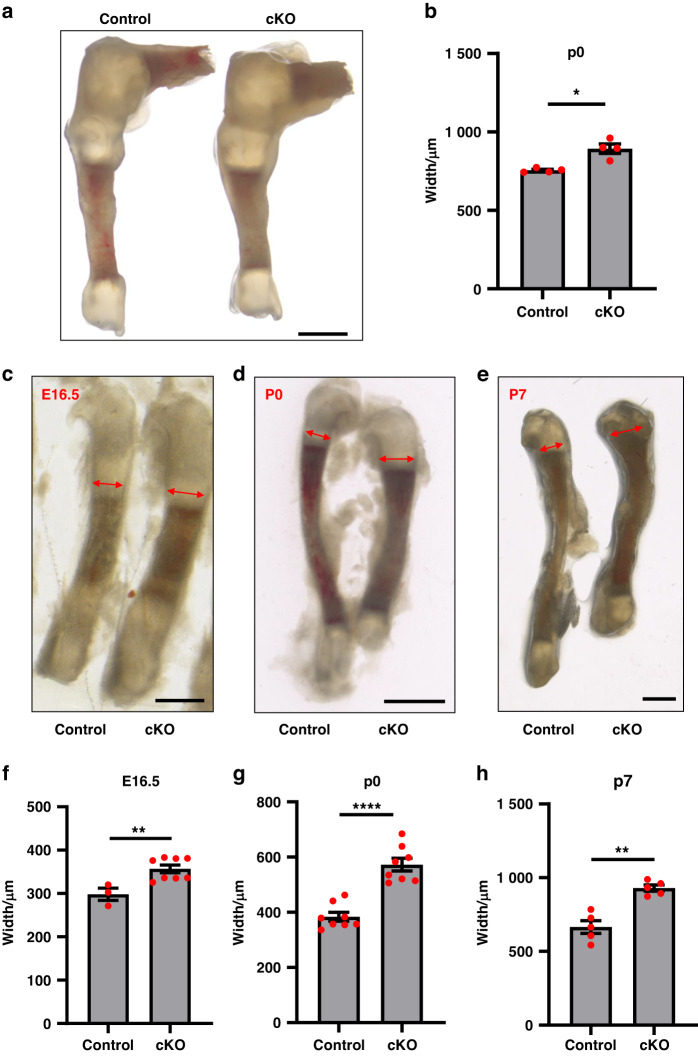
Loss of α-Parvin causes widening of long bones. **a** Stereoscopic image of knee joints of control and cKO mice at P0. Scale bar = 1 mm. **b** Comparison of the tibia width between control and cKO group, *P < 0.05. paired t-test. mean ± s.e.m. *n* = 4 mice for each group (**c**–**e**) Stereoscopic images of tibia from E16.5, P0, P7, and P30 control (left) and cKO mice (right). Scale bars = 500 μm, 500 μm, 1 mm respectively (from left to right). Double headed arrows denote the width of fibula. **f** Comparison of fibula width between control and cKO embryos at E16.5. **P < 0.01. unpaired t-test. mean ± s.e.m. *n* = 3 control and 8 cKO embryos from the same litter. **g** Comparison of fibula width between control and cKO mice at P0. ****P < 0.000 1. paired t-test. mean ± s.e.m. *n* = 8 mice for each group. **h** Comparison of fibula width between control and cKO mice at P7. **P < 0.01. paired t-test. mean ± s.e.m. *n* = 5 mice for each group

We next analyzed the width of the long bones and the growth plate cartilage of the cKO mice. In the tibia, measured at the chondro-osseous junction, we found a 15% increase in width compared to control at P0 (Fig. 2a, b). Interestingly, the fibula, a thin long bone that runs aside the tibia, behaved pronounced widening in cKO mice compared to control (Fig. 2c–h). Even at E16.5, when the difference in length was not yet evident, the fibula of cKO pups was already 15% wider than control (Fig. 2c, f). The difference in width further increased to 50% at P0 (Fig. 2d, g). The widening phenotypes diminished at P7, but a 30% increase could still be observed in the fibula of cKO pups compared to control (Fig. 2e, h). These data suggest that loss of α-parvin leads to excessive horizontal growth of long bones at the embryonic and neonatal stages.

### Loss of α-parvin causes disorganization of the growth plates

**Fig. 3:**
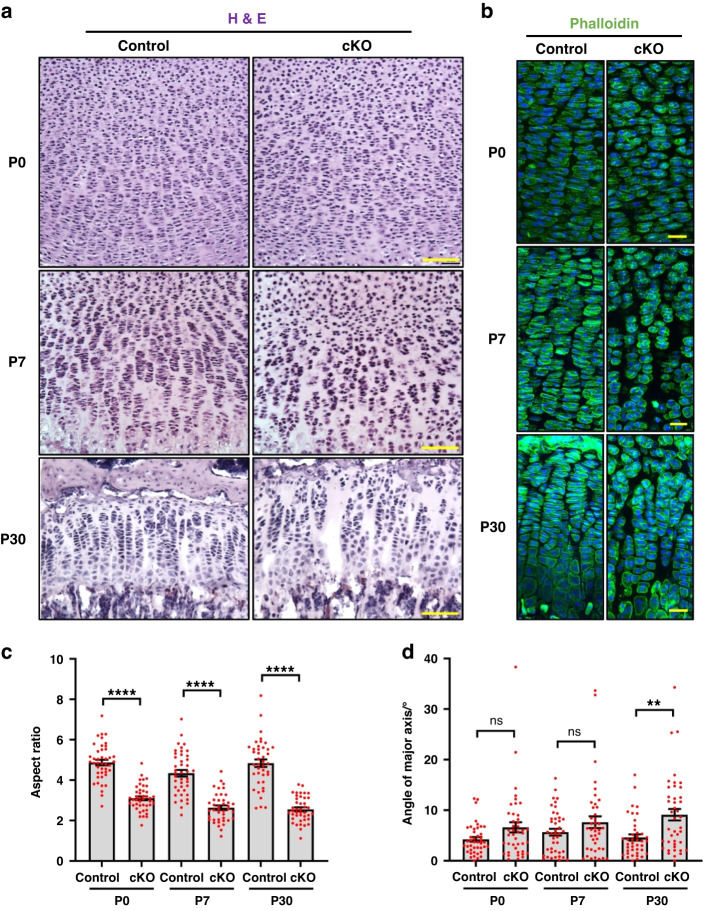
Loss of α-Parvin causes disorganization of growth plates. **a** H&E staining of the proximal tibia growth plate sections from control and cKO mice at P0, P7, and P30. Scale bars = 100 μm. **b** Staining of actin cytoskeleton. Sections of the proximal tibia growth plate were stained with Phalloidin (green) and counterstained with DAPI (blue). Scale bars = 20 μm. Quantification of the aspect ratio (**c**) and orientation (**d**) of chondrocytes in the proliferative zones of control and cKO growth plates at P0, P7, and P30. *ns*, not significant. **P < 0.01. ****P < 0.000 1. unpaired t-test. mean ± s.e.m. *n* > 40 cells for each group

Analyses of the growth plates of cKO mice revealed strikingly disorganized columnar structures. At P0 and P7, chondrocytes in the proliferative zone were normally arranged into columns that consist of at least 5 cells in control growth plates (Fig. 3a, b). In contrast, multiple short columns with less than five cells were found in the proliferative zone of cKO growth plates (Fig. 3a, b). At P30, similar columnar disorganization was found in the proliferative zone of cKO growth plates (Fig. 3a, b). Unexpectedly, the resting zone of cKO growth plates became dilated, indicating possible expansion of the stem cell population (Fig. 3a, b). In all stages, chondrocytes in the proliferative zone of cKO growth plates were much less elongated than control, as shown by the smaller aspect ratio (Fig. 3c). However, the orientation of chondrocytes only slightly increased in the cKO group (Fig. 3d). Moreover, the proliferative zone of cKO growth plates exhibited increased aggregation of chondrocytes. Around 5% of chondrocytes were found within an aggregate (Fig. S1A, B). This is confirmed by immunostaining with antibodies for pan-cadherin, which showed significant increase of cell-cell junctions in the proliferative zones of the cKO growth plates (Fig. S1C, D). These results suggest that loss of α-parvin causes disorganization of the growth plate cartilage.

### α-parvin regulates chondrocyte rotation

**Fig. 4:**
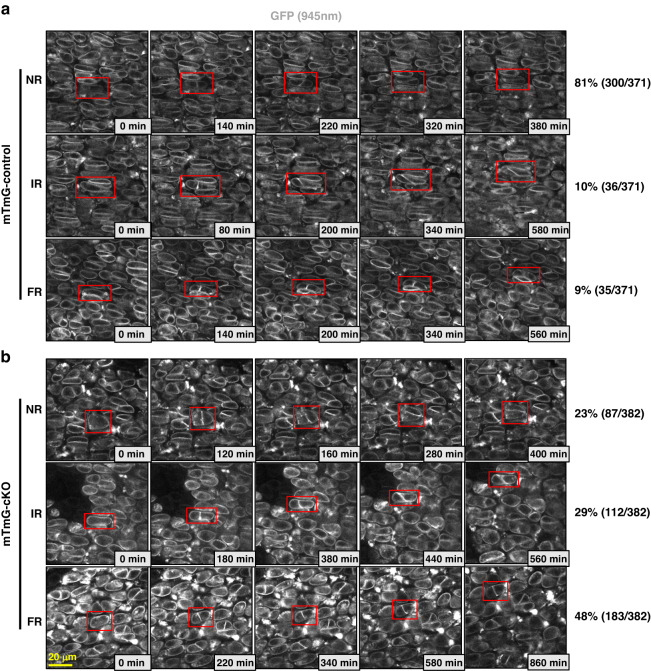
Live imaging of the growth plates in a fibula explant culture. **a**, **b** Snapshots from live imaging of chondrocytes undergoing chondrocyte rotation in mTmG-control or mTmG-cKO proximal fibula growth plates. The red rectangles highlight the dividing and rotating chondrocytes. NR normal rotation, IR incomplete rotation, FR failed rotation. GFP signals were excited with 945 nm laser. The boxes at the lower right of each image indicate the elapsed time. The percentages of each type of rotation events are shown on the right. The number of the corresponding type out of the total number of rotation events was shown in the brackets. In total, 371 rotation events of explant cultures from five mTmG-control mice and 382 rotation events of explant cultures from four cKO mice were counted. Scale bar = 20 μm

Live imaging of the growth plates is extremely valuable for monitoring chondrocytes’ behavior during long bone development. In chicks, the ex-vivo culture of the metatarsal or metacarpal has been used to study the mechanism of long bone growth and gene functions.^5,29,30^ In mice, Sarah et al. first applied live imaging on newborn pre-sphenoidal synchondrosis, a piece of tiny and flat cartilage in the cranial base, using *Rosa26*^*mTmG*^ *(mTmG)* transgenic mice.^31^ Keisho Hirota et al. also applied two-photon live imaging in culturing embryonic murine ulna to track the growth of long bones.^32^ To elucidate the cellular mechanism underlying the broader but shorter long bones of cKO mice, we employed two-photon live imaging and a neonatal fibula explant culture model to analyze the behavior of chondrocytes in situ. The fibula is a thin long bone that runs aside the tibia, with relatively flat growth plates. The surrounding tissues of the growth plates are thin, allowing two-photon microscopy of the chondrocytes. By combining two-photon live imaging and fibula explant culture, we could visualize chondrocyte rotation events within neonatal growth plates. In both control mice bearing mTmG reporters (mTmG-control mice) and *Rosa26*^*mTmG/+*^*; Prx1-Cre, Parva*^*flox/flox*^ mice (mTmG-cKO mice), consistent with the previous study,^31^ we found that the chondrocytes in the proliferative zone of fibula growth plates underwent a rotational movement process after cell division (Movies S1–S4). All daughter cells formed cell-cell adhesion after cytokinesis in both mTmG-control and mTmG-cKO group (Movies S1–S18). The daughter chondrocytes then pivoted along the cell adhesion surface and adjusted to the proper orientation, which could be parallel, orthogonal, or at a certain angle to the x-axis (chondro-osseous junction) (Fig. 4a, b, Movies S1–S18). We counted more than 300 events from at least three independent experiments for each group to quantify the chondrocyte rotation.

**Fig. 5:**
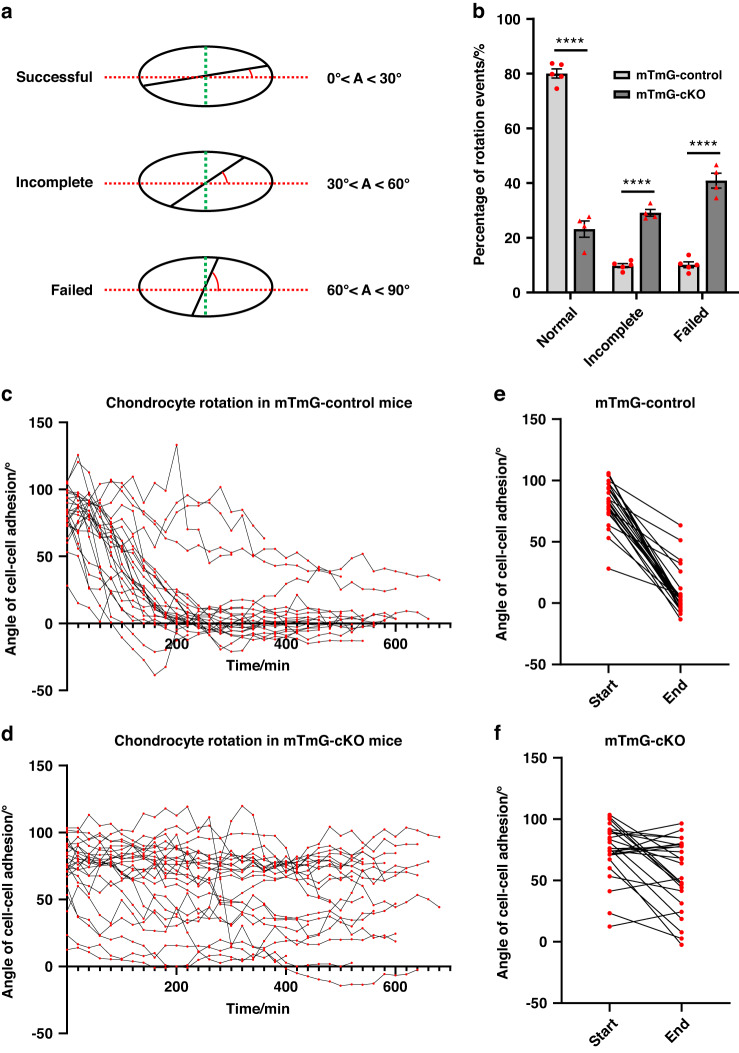
α-Parvin regulates chondrocyte rotation. **a** Classification of chondrocyte rotation events based on the final angles of cell-cell adhesion. Theoretically, the cell-cell adhesion bisects the major axis (green dashed line) after cytokinesis. Red dashed lines indicate the horizontal axis (x-axis) of long bones. **b** Summary of the outcomes of chondrocyte rotation events in mTmG-control and mTmG-cKO group. *n* = 5 explant cultures for mTmG-control group, *n* = 4 explant cultures for mTmG-cKO group. ****P < 0.000 1, unpaired t-test, mean ± s.e.m. Tracing of the angles of cell-cell adhesions during chondrocyte rotation in mTmG-control (**c**) and mTmG-cKO (**d**) group for up to 10 h. *n* = 25 rotation events for each group. **e**, **f** Angles of cell-cell adhesions at the beginning and at the end of the chondrocyte rotations. *n* = 25 rotation events for each group

In mTmG-control growth plates, most (81%) of chondrocytes normally rotated (NR), with a final orientation of cell-cell adhesion parallel or at an angle less than 30 degrees to the x-axis (Figs. 4a and 5a, b, Movies S1–S4). 10% of chondrocyte rotation events were incomplete (IR) in mTmG-control as the final angles of the cell-cell adhesion to the x-axis were more than 30 degrees (but less than 60 degrees) (Figs. 4a and 5a, b, Movie S5). 9% of mTmG-control chondrocytes did not rotate after cell division or stopped rotation at an angle greater than 60 degrees. These cells were classified as "Failed rotation" (FR) (Figs. 4a and 5a, b, Movie S6).

In contrast, in mTmG-cKO mice, a much larger percentage (48%) of growth plate chondrocytes failed to rotate (Figs. 4b and 5a, b, Movies S13–S16). Furthermore, incomplete rotation was observed in 29% of mTmG-cKO chondrocytes (Figs. 4b and 5a, b, Movies S9–S12). Only 23% of mTmG-cKO chondrocytes underwent normal rotation after cell division (Figs. 4b and 5a, b, Movies S7 and S8). By tracing the angles of cell-cell adhesion during the rotation process, we found that most chondrocytes in mTmG-control growth plates completed rotation within four hours, and the angles of cell-cell adhesion quickly converged to around 0° to the x-axis (Fig. 5c, e). Concomitantly, the daughter chondrocytes became flattened (Fig. 5c and Fig. S3A). By contrast, the angles of the cell-cell junction in most of the mTmG-cKO chondrocytes barely changed even after 10 hours (Fig. 5d, f). Since most mTmG-cKO chondrocytes did not undergo significant rotation, the flattening of the cells was also impaired (Fig. 5d and Fig. S3b). The net change of cell-cell adhesion angles also dramatically decreased to one-third of that in mTmG-control group (Fig. S3C).

Of note, 7% of mTmG-cKO chondrocytes were found to form aggregates during the live imaging experiments (Fig. S2A). One of the chondrocytes in a doublet divided before it separated from the sister cell. The doublet thus became a triplet and had three intercellular junction interfaces. They eventually adapted a three-pointed star style (Fig. S2A). Therefore, all three daughter cells failed to stack into the column. Indeed, mTmG-cKO growth plates contained chondrocyte aggregates within the proliferative zone, indicating its possible contribution to disorganized columns and wider long bones (Fig. S1A–D). By contrast, no aggregate formation was observed in mTmG-control chondrocytes. Taken together, with two-photon time-lapse imaging, our results suggest that α-parvin regulates chondrocyte rotation, which is necessary for the formation of the columnar structure.

### Loss of α-parvin induces multiple chondrocyte defects

**Fig. 6:**
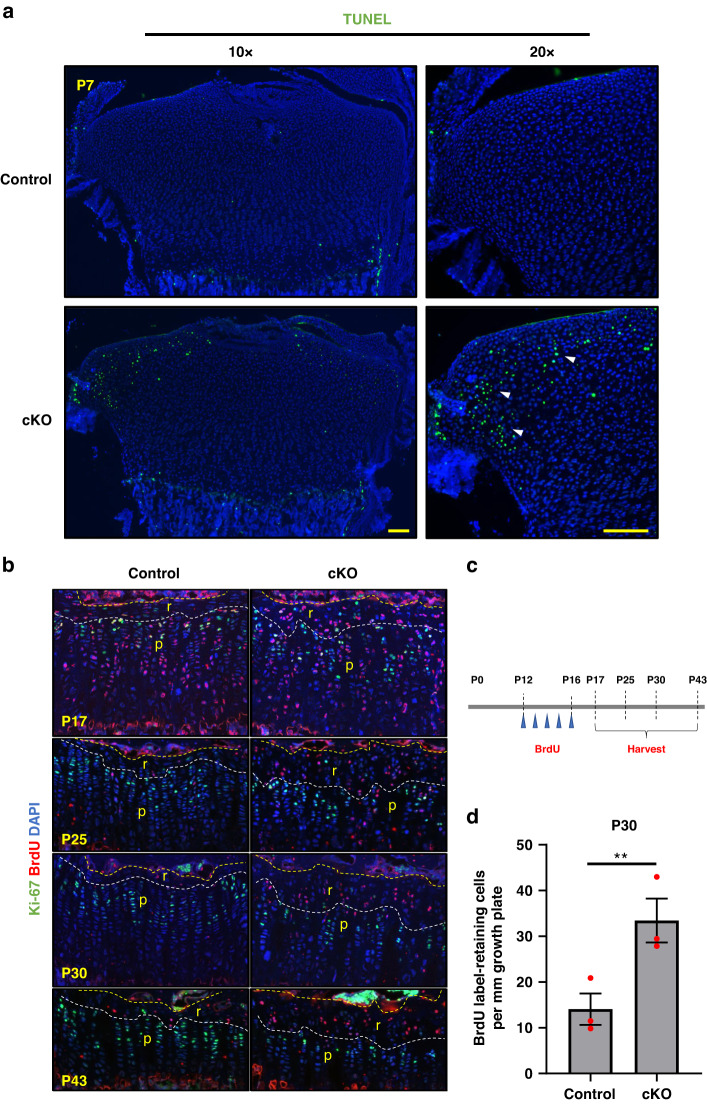
Loss of α-parvin results in increased apoptosis and dilated resting zone. **a** Apoptosis. Sections of the P7 proximal tibia growth plates were stained using TUNEL assay and counterstained with DAPI. Scale bars = 100 μm. **b** BrdU label retention assays. Sections of the proximal tibia growth plates at different stages were stained for BrdU and Ki-67 and co-stained with DAPI. r resting zone, p proliferative zone. Scale bars = 100 μm. **c** Experiment design of BrdU label-retention assays. Five doses of BrdU at 50 mg·kg^−1^ were administered on each day from P12 to P16. Mice were sacrificed at P17, P25, P30, and P43 and the bones were collected for analysis. **d** Quantification of the number of label-retaining cells (LRCs) per millimeter growth plate in control and cKO growth plates at P30. **P < 0.01. paired t-test. mean ± s.e.m. *n* = 3 mice for each group

In growth plate cartilage, apoptosis typically occurs within the hypertrophic zone and borderline area.^33^ To assess the effect of α-parvin deficiency on cell survival, we performed Terminal deoxynucleotidyl transferase dUTP nick end labeling (TUNEL) staining on control and cKO growth plates. Although no change of apoptosis was found between the control and cKO hypertrophic zone and the lower borderline area, we detected significant increase of apoptosis in the cKO growth plates compared to that of the control at P7 (Fig. 6a). Most TUNEL signals were detected in the lateral region of cKO resting zone (Fig. 6a). In addition to elevated cell death, we also found increased binucleation at the late neonatal stage (P5-P7) in cKO growth plates. The percentage of binucleated cKO chondrocytes was similar to that of the control at P0 but it was increased significantly (to 8%) at P5-P7 (Fig. S4E, F). BrdU incorporation assays showed that the percentage of BrdU-positive nuclei of cKO proliferative zones were comparable with that of control, indicating that the entry into S phase was unaffected in cKO chondrocytes at the neonatal and embryonic stages (Fig. S4A–D).

Previous studies have shown that mature or adolescent resting zone of growth plates harbor long-term progenitor cells, which is different from neonatal growth plates.^3,4^ We found no difference between neonatal cKO and control resting zones as for the shape and number of chondrocytes. However, the resting zone of mature cKO growth plates became significantly thicker than that of the control (Figs. 6b and 3a, b). To test if the chondroprogenitors increased in the resting zone of cKO growth plates, we carried out BrdU label retention assay, which marks putative stem cells (Fig. 6c).^4,34^ We detected more label-retaining cells (LRCs, putative stem cells) in mature cKO growth plates compared to that of the control (Fig. 6b, c). The resting zones of control growth plates were less than 50 μm in thickness from P17 to P43, while the thickness of cKO growth plates was around 100 μm. At P30, the number of LRCs in cKO growth plates doubled compared to that of control (Fig. 6d). These results demonstrate that the resting zone of mature cKO growth plates becomes dilated, implying a possible role of α-parvin in regulating skeletal stem cell niches.

### Single-cell RNA-seq analysis of growth plate chondrocytes

To determine the effects of α-parvin deficiency on the transcriptome of chondrocytes in each distinct zone within the growth plates, we performed single-cell RNA-seq (scRNA-seq) analyses of the control and cKO growth plates. The Uniform Manifold Approximation and Projection (UMAP) dimension reduction and clustering of scRNA-seq data successfully grouped the cells into several distinct zones of the growth plates (i.e., hypertrophic zone, resting zone, proliferative zone, and perichondrium) (Fig. S5A). Next, we analyzed the differentially expressed genes (DEGs) for each cluster. The results showed that gene expression was altered in all three distinct zones of the growth plates (Fig. S6, Table S1). Within the DEGs, some have been reported to regulate chondrogenesis/hypertrophy. Specifically, *Tgm2* and *Fxyd2*, the positive regulators of chondrocyte maturation, increased in the cKO hypertrophic zone (Cluster 0) (Fig. S5B). The marker of hypertrophic chondrocytes, *Col10a1*, also elevated moderately in the lower cKO proliferative zone (Cluster 1) (Table S1). *Ccn2*/connective tissue growth factor (CTGF), a multifaceted regulator of skeletal growth, was significantly downregulated (75% and 50% decrease in the lower resting zone and upper proliferative zone) in both the proliferative and resting zones (Clusters 1, 2, 4, 5, 7, and 8) of cKO growth plates (Fig. S5B). Additionally, we noticed an upregulation of osteoarthritis/inflammation-related genes (e.g., *Ier3*, *Klf4*, *Cytl1*) and a downregulation of chondroprotective genes (e.g., *A2m*, *C1qtnf3*, *Matn1*, *Matn3*)^35–40^ in the cKO growth plates (Fig. S5B). Gene ontology analysis of DEGs from the upper proliferative zone (Cluster 2) suggests significant enrichment in cell-matrix adhesion, cytoskeleton, and ECM (Fig. S7). Indeed, several genes (*Ezr*, *Epyc*, *Enah*, *Dcn*, *Cd44*, *Actg1*, *Dstn*) of these GO terms were altered significantly (Fig. S5B). However, there was no enrichment in other classical pathways of skeletal growth, such as Fgf, Ihh, and Bmp signaling. These data suggest an altered transcriptome of mutant chondrocytes in multiple zones of the growth plates, providing an explanation of at least some aspects of the observed chondrodysplasia phenotype in the cKO mice.

## Discussion

### Live imaging of the growth plates reveals an important role of α-parvin in chondrocyte column formation

**Fig. 7:**
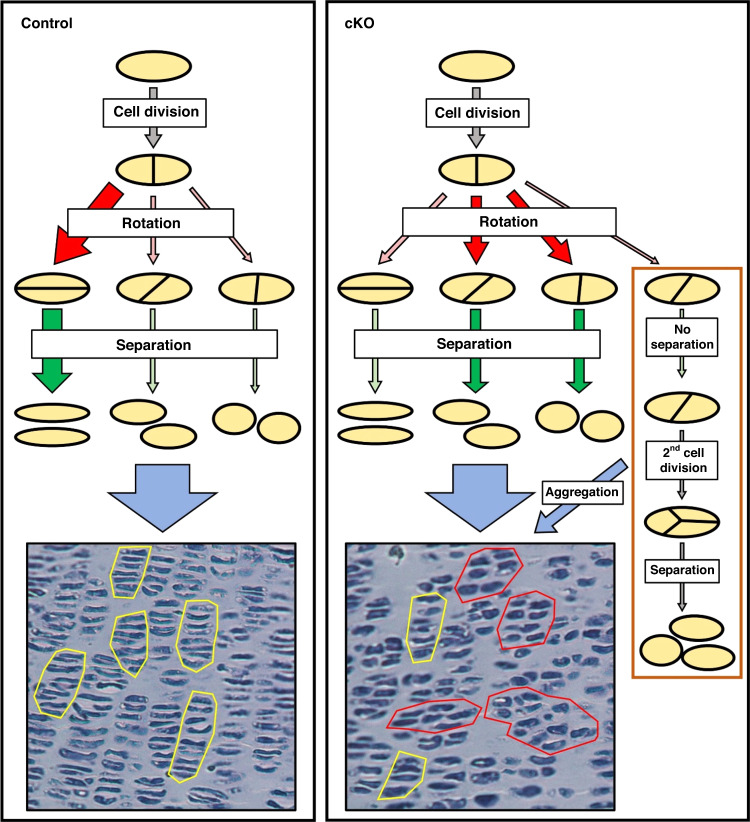
The roles of α-parvin in chondrocyte column formation. The picture depicts a model in which loss of α-parvin impairs long bone development. In control group, most chondrocyte rotations are successful (red arrow). The daughter cells become flattened after rotation. After separation, the daughter cells of control group stack on top of each other (green arrow) into columnar structures, as shown by the H&E staining (left bottom panel). However, in cKO group, most of the rotation events are unsuccessful (incomplete or failed) (red arrows), and the cKO chondrocytes form aggregates (brown rectangle). Both defective rotation and aggregate formation lead to disorganized columnar structures after separation (right bottom panel), resulting in unbalanced longitudinal and horizontal growth

In the current study, we have found that loss of α-parvin causes aberrant shortening and widening of long bones with disorganized proliferative zone, illustrating a crucial role of α-parvin in long bone development. Furthermore, we have investigated the cellular mechanism by which α-parvin functions in this process. Column formation is a characteristic feature of chondrocytes in the proliferative zone of growth plates that is crucial for long bone development. This process is likely accomplished by well-orchestrated chondrocyte division, rotation, and daughter cell separation. Using a combination of two-photon live imaging and fibula explant culture, we show that α-parvin is required for chondrocyte rotation. Based on dynamic imaging of growth plate chondrocytes, we analyzed the rotation of chondrocytes, which was classified as “normal”, “incomplete” or “failed”, in both mTmG-control and mTmG-cKO group (Fig. 7). The results showed that α-parvin-deficient chondrocytes were prone to fail in chondrocyte rotation, and the daughter cells were often unable to elongate their major axis properly as compared to those in the mTmG-control group (Fig. 7). In addition, cKO chondrocytes formed more aggregates in the proliferative zone, possibly due to the delayed separation of daughter chondrocytes. Both unsuccessful rotation and aggregation of chondrocytes likely place the daughter cells laterally (rather than stacking into columns) after separation (Fig. 7). Based on the studies described in this paper, we propose a model in which loss of α-parvin causes unsuccessful rotation and aggregation of chondrocytes, which might unbalance the transverse and longitudinal growth of the growth plate cartilage, resulting in shorter but broader long bones in cKO mice (Fig. 7). In addition, these results suggest that fibula is a suitable ex-vivo model to conduct live imaging and study the cellular behaviors of the growth plate chondrocytes.

How does α-parvin regulate chondrocyte rotation and aggregation? Rearrangement of cells is driven by the anisotropy that is generated through adhesion, surface tension, and cytoskeleton.^31,41–43^ Previous research has suggested crucial roles of the cytoskeleton, cell-ECM adhesion, and cell-cell adhesion in regulating chondrocyte rotation.^5,31^ Notably, loss of β1 integrin also causes severe chondrodysplasia and retards the growth of long bones.^13^ Given the similar phenotypes in α-parvin cKO growth plates and integrin β1 cKO growth plates, it is conceivable that α-parvin may regulate rotation through influencing integrin signaling, cytoskeleton, and cell-ECM adhesion.^18,21^ In addition, loss of α-parvin may also alter cell-cell adhesion (Supplementary Fig. S1). Collectively, the alterations in the cytoskeleton, cell-cell, and cell-ECM adhesion may delay the separation step of column formation and result in defects in chondrocyte rotation and aggregation.

### Loss of α-parvin induces multiple defects of chondrocytes that contribute to defective skeletal growth

In addition to the abnormal chondrocyte rotation, other defects of chondrocytes may also contribute to the dwarfism of cKO mice. We have found that loss of α-parvin increased the percentage of binucleated chondrocytes, which is consistent with previous studies showing that loss of profilin-1 or integrin β1 causes defective cytokinesis in the growth plates.^13,44^ However, despite of our efforts, we were unable to detect α-parvin in mitosis-related structures (e.g., mitotic spindle, centrosome, contractile ring, and midbody) and did not observe any obvious defects in the midbody structure of cKO chondrocytes (data not shown). Thus, α-parvin probably affects cytokinesis indirectly, possibly through influencing the integrin and cytoskeletal pathways. In addition to the effect on binucleation, we have found a significant increase of apoptosis in the lateral region of the resting zone in cKO growth plates, suggesting a crucial role of α-parvin in protecting this population of cells from cell death. It will be interesting to determine to what extent this aberrant cell death contributes to defective long bone growth.

It is interesting to compare the phenotype of the α-parvin cKO mice described in the current study with that of the ILK cKO mice, in which the ILK gene in chondrocytes was inactivated using Cre recombinase under the control of the collagen II promoter.^45,46^ Similar to what we have found in the α-parvin cKO mice, loss of ILK in the chondrocytes resulted in disorganization of the columnar arrangement of chondrocytes in the growth plates and dwarfism.^45,46^ Although it remains to be determined experimentally, our finding that loss of α-parvin impairs chondrocyte rotation (Figs. 4 and 5) suggests that the disorganized columnar arrangement of chondrocytes observed in the ILK cKO mice^45,46^ may also result from the defect in chondrocyte rotation. While the phenotypes of the α-parvin cKO and the ILK cKO mice share considerable similarities, they are not identical. For example, while all the ILK cKO mice died within day 1 after birth,^45^ no death of the α-parvin cKO mice was observed until day 35 after birth. Thus, while it is likely that α-parvin and ILK work in concert in chondrocyte rotation and columnar organization in growth plates and long bone development, they may possess functions that are independent from each other in other processes. Alternatively, this may reflect the difference in the promoters (Prx1 vs. collagen II) that were used to drive Cre expression during generation of the α-parvin cKO and ILK cKO mouse lines.

Finally, mature growth plates harbor long-term chondroprogenitors in the resting zone.^3,4,47–49^ Besides the columnar disorganization in mature cKO growth plates, we also found that the resting zone of cKO growth plates became dilated and contained more stem cells, indicating the importance of α-parvin in regulating the maintenance of chondroprogenitors. Of note, integrin signaling is known to be crucial for regulation of stem cells in a number of systems.^50^ For example, ablation of β1-integrins in intestinal epithelium resulted in hyperproliferation of crypt stem cells, likely through downregulation of Hedgehog signaling.^51^ In the subventricular zone, blocking α6β1 integrins caused increased proliferation of subventricular zone neural stem cells.^52^ Given the fact that α-parvin is a prominent component of the integrin signaling pathway, it is conceivable that α-parvin may regulate the behavior of chondroprogenitors in the growth plate through influencing integrin signaling. It will be interesting to test this possibility and determine the mechanism by which α-parvin regulates the growth plate stem cells in future studies.

### Implications from transcriptomic analysis of the growth plates

scRNA-seq provides a map of transcriptomic changes in different cell types. Using this experimental approach, we have found alterations of transcriptome in all three zones (i.e., resting, proliferative, and hypertrophic zones) within the cKO growth plates. For example, we have detected an increase of hypertrophy-related genes in the hypertrophic zone and lower proliferative zone of cKO growth plates, indicating a possible acceleration of chondrocyte differentiation. Interestingly, a group of osteoarthritis-correlated genes was upregulated, and several chondroprotective genes were downregulated in cKO resting zone, which could explain the increased cell death in the resting zone of cKO growth plates. However, among all the DEGs that have been studied in skeletal growth, none of them have been shown to directly control polarity or column formation. GO analysis showed altered expression of genes from adhesion, ECM components, and cytoskeleton. Since the chondrocyte rotation likely relies on the anisotropy established by adhesion and cytoskeleton, these data raise a possibility that α-parvin might also affect chondrocyte rotation through transcriptional regulation. Due to the lack of appropriate in-vitro models, we could not directly test the contribution of each DEG to the chondrocyte rotation defects in the current study. Nevertheless, the scRNA-seq analyses of the cKO growth plates have laid foundation for future studies aimed at determining the functions of α-parvin-mediated transcriptional regulation in different zones of the growth plates during long bone development.

In conclusion, the studies presented in the current paper have demonstrated that α-parvin is indispensable for long bone development. Loss of α-parvin leads to increased apoptosis and cytokinesis defects and causes dilation of the resting zone of growth plates. Importantly, our studies have revealed a crucial role of α-parvin in regulating chondrocyte rotation, aggregation, and column formation, which we propose contributes to the imbalance between the transverse and longitudinal growth in the cKO mice, resulting in defects in the neonatal long bone development.

## Methods

### Mice

The experiments have been approved by Institutional Animal Care and Use Committee of the Southern University of Science and Technology. *Prx1-Cre* mice (Stock No: 005584) were purchased from Jackson Laboratory. *Parva*^*flox*^ mice were generated by Shanghai Biomodel Organism Science & Technology Development Co. Ltd and maintained under C57/BL6j background. Two LoxP sequences were inserted to flank exon 2 and exon 3 of *Parva*. *Rosa26*^*mTmG*^ (*mTmG*) mice (Stock No: 007676) were gifts from Dr. Shengjian Ji, SUSTech.

For the analysis of bone phenotypes, we generated limb bud-specific α-parvin cKO mice, Briefly, *Parva*^*flox/flox*^ mice (F0) were crossed with *Prx1-Cre* mice (F0) to obtain *Parva*^*flox/+*^*; Prx1-Cre* mice (F1). *Parva*^*flox/+*^*; Prx1-Cre* males (F1) were then bred with wild-type females to obtain *Parva*^*flox/+*^*; Prx1-Cre* mice whose *Parva*^*flox*^ and *Prx1-Cre* alleles putatively localized in the same chromosome (F2). Males of these *Parva*^*flox/+*^*; Prx1-Cre* mice (F2) were then crossed with *Parva*^*flox/flox*^ females or *Parva*^*flox/+*^ females to obtain *Parva*^*flox/flox*^*; Prx1-Cre* mice (F3) (cKO mice). All the cKO mice were alive until day 35 after birth, when they began to die and all of them died before two months after birth. *Parva*^*flox/+*^*; Prx1-Cre, Parva*^*flox/flox*^*, Prx1-Cre, Parva*^*flox/+*^, or *wild-type* mice were phenotypically normal and used as control mice.

For time-lapse imaging of the bone explant culture, *Parva*^*flox/flox*^ mice were crossed with *Rosa26*^*mTmG/mTmG*^ mice to generate *Parva*^*flox/+*^*; Rosa26*^*mTmG/+*^ mice. *Parva*^*flox/+*^*; Rosa26*^*mTmG/+*^ females were then crossed with *Parva*^*flox/+*^*; Prx1-Cre* males to generate *Parva*^*flox/flox*^*; Prx1*^*Cre*^*; Rosa26*^*mTmG/+*^ mice. *Parva*^*flox/+*^*; Prx1*^*Cre*^*; Rosa26*^*mTmG/+*^, *Parva*^*flox/+*^*; Rosa26*^*mTmG/+*^, *Parva*^*flox/flox*^*; Rosa26*^*mTmG/+*^, or *Prx1*^*Cre*^*; Rosa26*^*mTmG/+*^ mice were used as mTmG-control mice.

### Histology

The tibia, fibula, and femur head of neonatal or adolescent mice were dissected using fine forceps and scissors. Samples were immersed in 2–5 mL 4% PFA and allowed to fix for 20 h at room temperature or 4 °C. Bones were then decalcified in 0.5 mol·L^−1^ EDTA pH8.0 at room temperature for 2–14 days, depending on the age of mice (2 days for bones of P0 and E16.5, seven days for bones of P7, and 14 days for bones of P30 or elder mice).

Decalcified bones were then subjected to paraffin section or cryosection. For paraffin sections, decalcified long bones were dehydrated through alcohol gradient, xylene, and embedded in paraffin. The paraffin sections were cut at 5μm and stored at 4 °C. For cryosection, decalcified bones were dehydrated in 30% sucrose in PBS for 2–5 days and equilibrated in OCT 30% sucrose 1:1(v/v) mixture for several hours before embedding. 8 μm Sections were prepared with Leica Cm1950 Cryostat and stored at −20 °C before use.

### HE staining

Hematoxylin-eosin staining was performed on paraffin sections. After deparaffinization and rehydration, paraffin sections were stained with hematoxylin for 5 min, then immersed in 70% ethanol with 0.5% hydrochloric acid for 5 s. Sections were then rinsed in tap water for 0.5–1 h. Eosin staining was carried out for 1 min. After washing with distilled water, slides were dehydrated and mounted. Images were then acquired using a Nikon upright microscope (Eclipse Ni-U) equipped with a digital camera.

### BrdU incorporation assay and detection

To assess chondrocyte proliferation, P0, P7 mice, and E16.5 pregnant mice were administered with BrdU (Sigma B5002) at 50 mg·kg^−1^ intraperitoneally, except for P0, for which mice were injected with BrdU subcutaneously. All mice were sacrificed 3 h after administration.

BrdU label retention assay was performed by administering one dose of BrdU each day at 50 mg·kg^−1^ between P12-P16,^3^ and the mice were sacrificed either at P17, P30, or P43 to evaluate the BrdU label-retaining cells (i.e., possible chondroprogenitor).

The following pretreatment methods were used to visualize BrdU on tissue sections. After rehydration with PBS, sections were rinsed in ddH2O and PBS. After that, sections were permeablized with 1% Triton-X 100 in PBS for 15 min and rinsed in PBS for 3 × 5 min. Antigen retrieval was carried out by incubating the slides with 0.01 mol·L^−1^ citric acid pH6.0 at 60 °C overnight. On day2, after cooling to room temperature, slides were rinsed in PBS several times and then treated with 1 mol·L^−1^ HCl for 30 min at 37 °C. Droplets of sodium borate pH8.5 were added onto the sections for 3 × 10 min to neutralize the hydrochloric acid. After that, sections were subjected to immunostaining with a monoclonal BrdU antibody from Proteintech (Cat No. 66241).

### Immunostaining

To prepare the cytosmear of primary chondrocytes, freshly isolated cells from the proximal tibia growth plates were fixed in 4% PFA for 15 min, and then briefly spun down. The cell pellets were washed three times with PBS and three times with ddH2O. Then a droplet of cell suspension was added onto glass slides and allowed to dry at room temperature. Slides were stored at −20 °C before use.

Immunostaining was performed using cytosmears and frozen sections. Briefly, frozen sections or cytosmears were rehydrated in PBS, followed by permeabilization in 1% Triton-x 100 in PBS for 15 min. After rinsing in PBS for 3 × 5 min, slides were blocked by 10% normal goat serum containing 0.1% Triton-x 100 in PBS for 1 h, then incubated with primary antibodies overnight. For pan-cadherin and Ki-67 staining, an additional antigen retrieval step was performed by incubating the slides with antigen retrieval buffer (citric acid pH6.0) at 60 °C overnight. After that, cytosmear or sections were rinsed in PBS several times and incubated in the blocking buffer containing DAPI and secondary antibodies or phalloidin for 1 hr at room temperature. Finally, slides were rinsed in PBS 6 × 5 min and then mounted with Vectashield anti-fade mounting medium (H-1200-10) or mixture of glycerol and PBS (50:50). Sections and cytosmear were scanned by laser scanning microscope (Leica, Sp8). Antibodies and reagents used in staining were as follows, phalloidin (Invitrogen, A12379, 1:500), mouse monoclonal anti-pan-cadherin (Santa Cruz, sc-59876, 1:200), rabbit anti-Ki-67 (Cell Signaling Technology, #12202, 1:500), rabbit anti-α-parvin (Cell Signaling Technology, #8190, 1:200).

TUNEL staining was performed with In situ Cell Death Detection Kit (Roche, #12156792910). Briefly, frozen sections were dehydrated and incubated with 5 μg·mL^−1^ Proteinase K (Takara #9034) diluted in PBS for 30 min at 37 °C. After washing 6 × 5 min in PBS, sections were immersed in reaction solution. Finally, slides were washed with PBS for 6 × 5 min and mounted.

### Live imaging of murine growth plates

The fibula of P0-P1 pups was used for explant culture and live imaging. Briefly, the fibula was first dissected out. Muscles and tibia were carefully removed with ultrafine forceps under a stereoscope. The perichondrium/periosteum of the fibula was not removed to minimize the damage to the growth plate microenvironment. Then, the fibula was immobilized by a pre-made mold of 1% agarose gel in a glass-bottom dish (#801001, Wuxi, *Nest*). 2 mL medium was added, and the culture was equilibrated at 37 °C 5% CO_2_ for 2 h. The medium composition is as follows: α-MEM (41061-029) supplement with 0.3% BSA, 50 μg·mL^−1^ L-ascorbic acid, 10 mmol·L^−1^ β-glycerophosphate, 10 nmol·L^−1^ β-mercaptoethanol, and antibiotics.

For two-photon live imaging, we used an Olympus FVMPE-RS inverted two-photon microscope. 1040 nm and 945 nm lasers were used to excite tdTomato and eGFP, respectively. For both wavelengths, the laser power was set to 5%–9% (automatically adjusted with increased depth of imaging by the software). Since the GFP signal was brighter than tdTomato, the HV value for the NDD detector was set to 500 for eGFP and 540 for tdTomato. Approximately 150 μm thick proliferative zone with part of the hypertrophic zone and resting zone of the fibula growth plate was imaged every 20 min. The thickness of each plane was 1.5 μm. The resolution was set to 1 024 × 1 024 (424 μm × 424 μm). The culture was imaged for 16–24 h in a stage-top incubator with passive humidity at 37 °C and 5% CO_2_ atmosphere.

Data were exported and subjected to analysis with ImageJ software. Videos recording the chondrocyte rotation events were cropped.

### scRNA-seq and analysis

For scRNA-seq, proximal tibial growth plates of littermates aged P6 were digested with 0.2% type II collagenase for 3 h. The cells were then subjected to scRNA-seq with BD rhapsody for library construction. The scRNA-seq data was analyzed by R with Seurat package.^53^ Briefly, raw expression matrices were imported into Rstudio software. The cells with more than 5% mitochondrial genes and less than 200 features were removed. The cells with high expression of heat-shock protein were also removed before downstream analysis. The data from cKO and control group were then normalized with SCTransform and integrated.^54,55^ Dimension reduction and clustering were performed on the integrated data with resolution set to 0.7. The cells in different clusters were annotated based on cell-type-specific markers. The DEGs were generated by comparing control and cKO chondrocytes in each cluster (e.g., cKO Cluster1 vs control Cluster1, cKO Cluster0 vs control Cluster0). GO analysis was conducted with gProfileR2 package.^56^

### Statistical analysis

Statistical analysis was performed using Student’s *t* test with GraphPad Prism 8. Data are presented as Mean ± SEM. A *P*-value < 0.05 was considered significant.

### Supplementary information

Chondrocyte rotation in control and cKO growth plate chondrocytes

Supplementary figures and tables
