# Supplementary material for: α-parvin controls chondrocyte column formation and regulates long bone development
Source: Bone Res. 2023 Aug 22;11:46. doi: 10.1038/s41413-023-00284-7 (PMC10444880; doi:10.1038/s41413-023-00284-7)
Supplement: Supplementary file 2 — Supplementary figures and tables [file 41413_2023_284_MOESM2_ESM.docx]

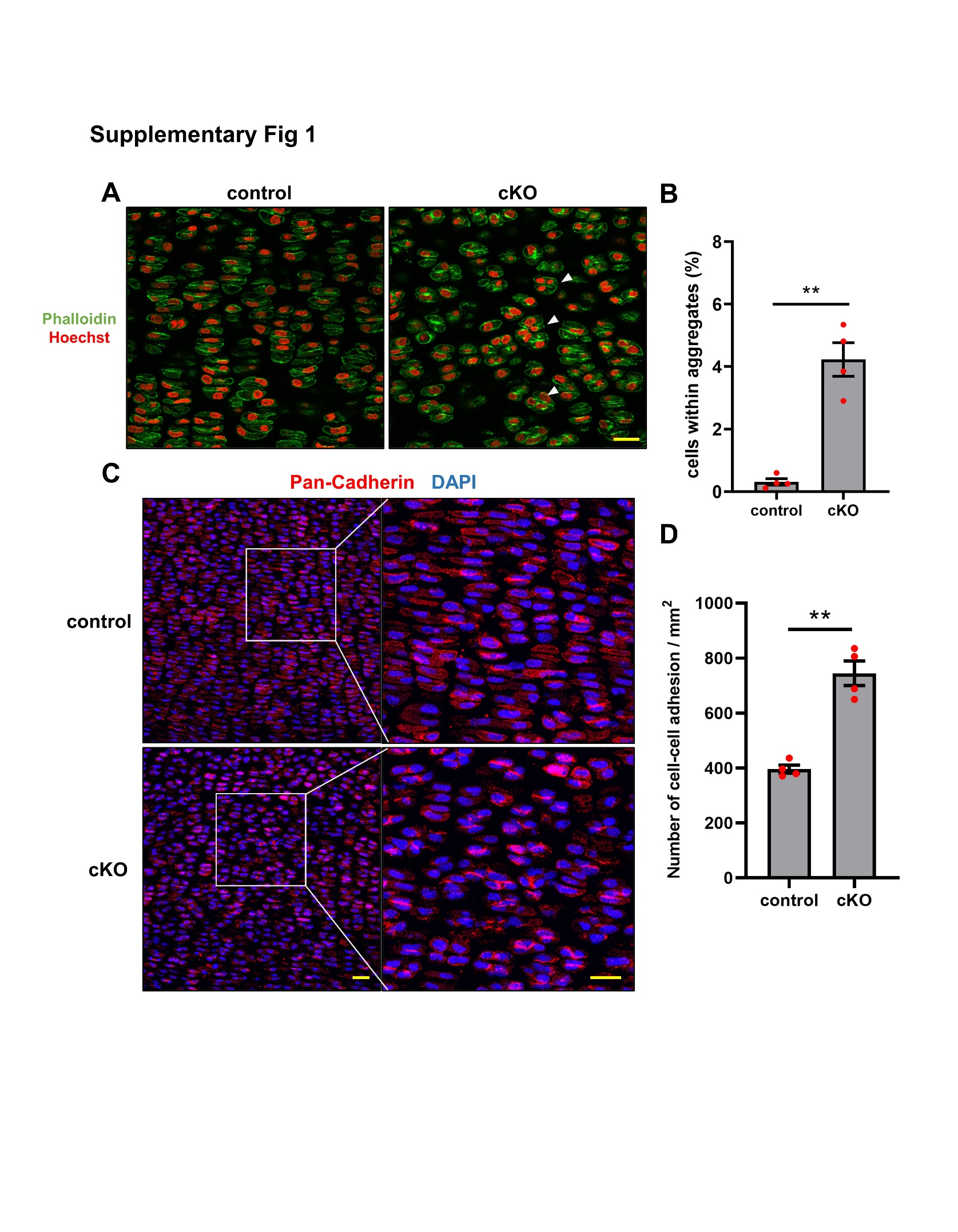
**Supplementary Information**

## Supplementary Figure 1. Aggregation of cKO growth plate chondrocytes

(A) Phalloidin staining (green) of the proximal tibia growth plates of control and cKO group at P0, counterstained with Hoechst 33342 (red). Scale bar = 20 μm. (B) Quantification of aggregates on the sections of proximal tibial growth plates stained with phalloidin. **, *p*<0.01. paired t-test. mean±s.e.m. n = 4 mice for each group. (C) Detection of cell-cell adhesions. Sections of the proximal tibia growth plates from P0 control and cKO mice were stained for pan-cadherin (red) and counterstained with DAPI (blue). Scale bars = 20 μm. (D) Quantification of the number of cell-cell adhesions in the proliferative zones of control and cKO growth plates. **, *p*<0.01. paired t-test. mean±s.e.m. n = 4 mice for each group. For each data point, the number of cell-cell adhesions from one growth plate proliferative zone was counted and divided by the area of corresponding proliferative zone.

##
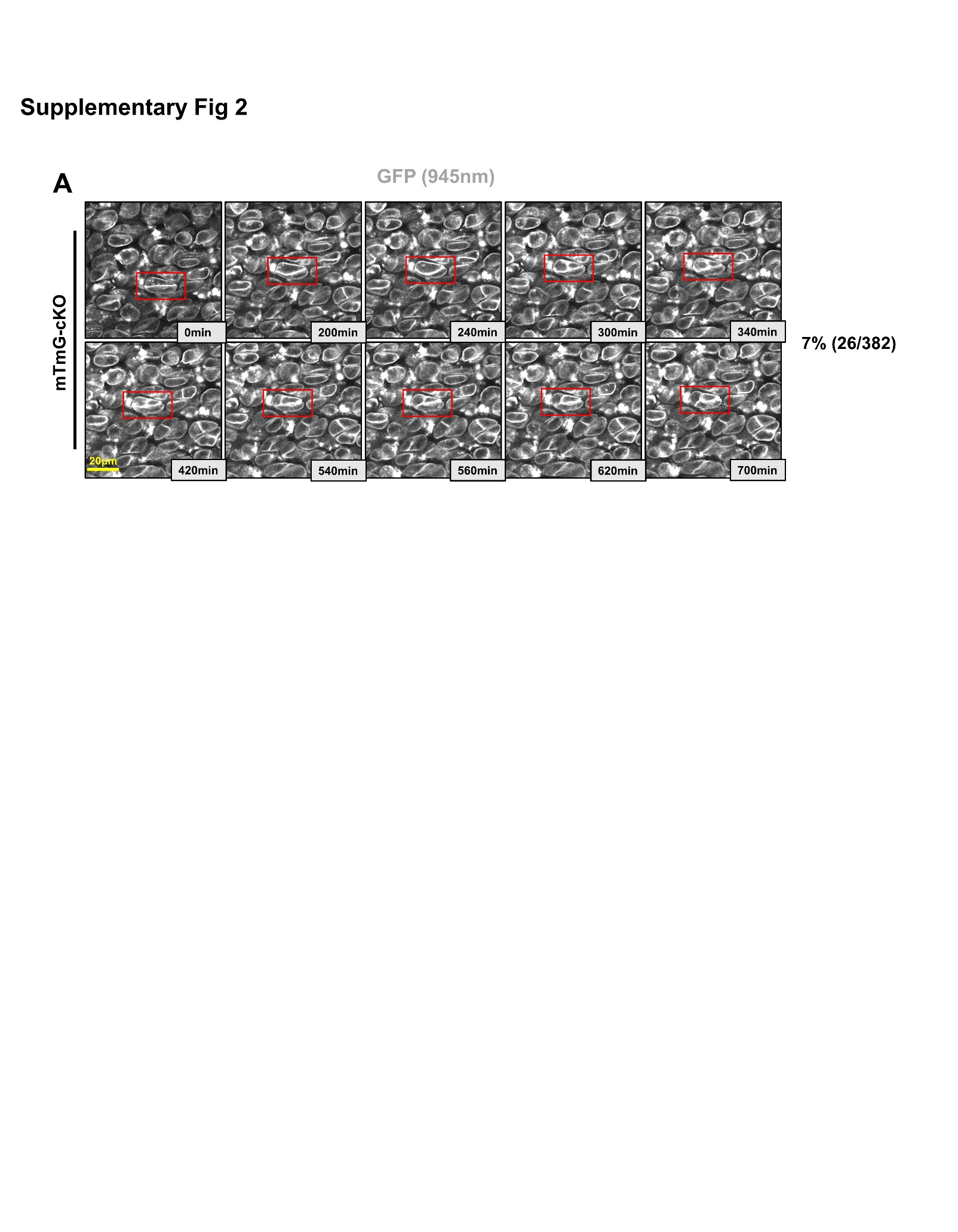
Supplementary Figure 2. Formation of chondrocyte aggregates in live imaging experiments

(A) Two-photon live imaging of mTmG-cKO growth plates. The red rectangles highlight the chondrocyte doublets that underwent second division before they could fully separate from each other, leading to triplet structure. Scale bar = 20 μm. GFP signals were excited by 945nm laser. The box at the lower right of each image indicates the elapsed time. The percentages of aggregate formation are shown on the right. The number of aggregations out of the total number of rotation events was shown in the brackets.

##
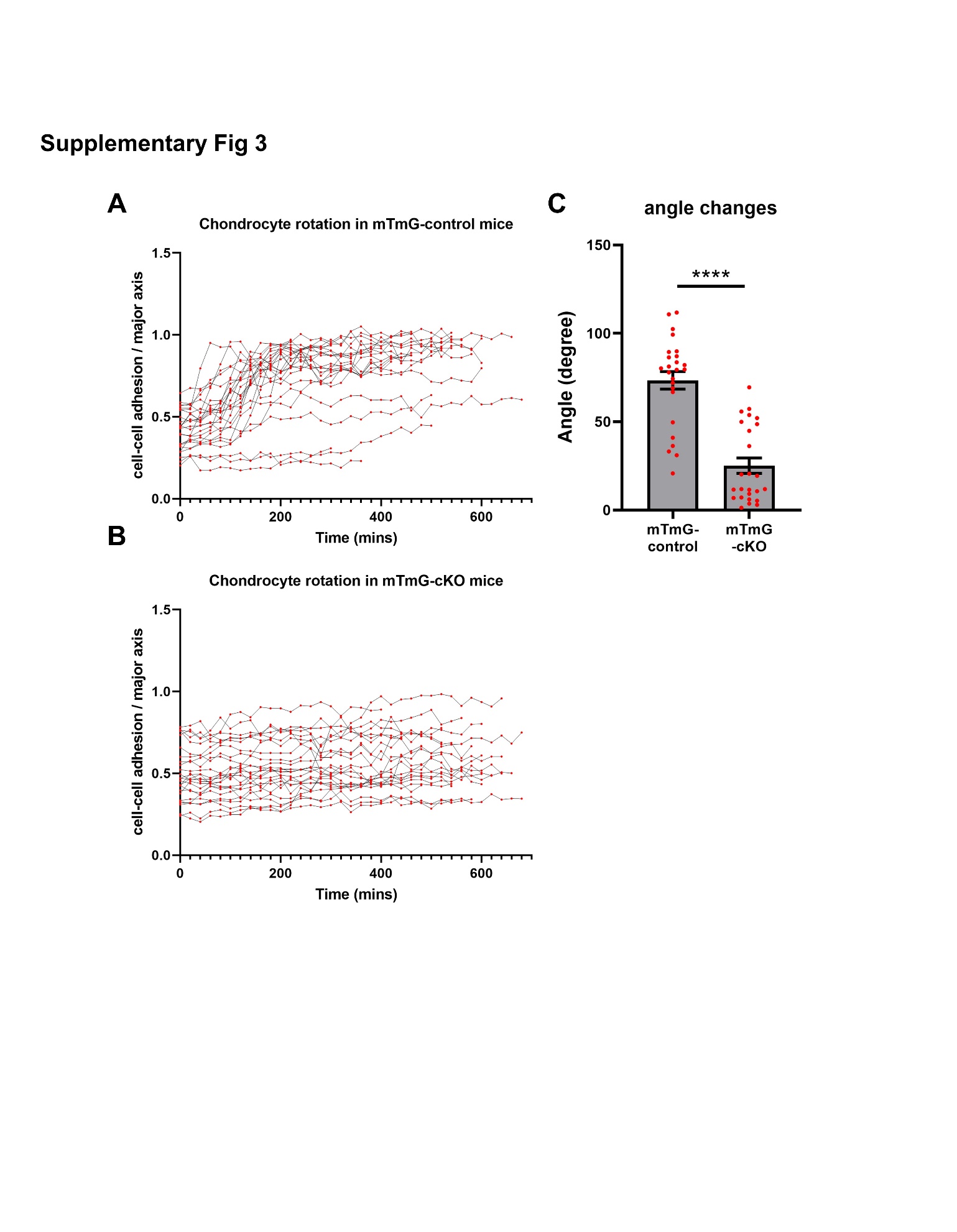
Supplementary Figure 3. Quantification of chondrocyte rotation

(A-B) Flattening of growth plate chondrocytes during rotation. The ratio of the length of cell-cell adhesion to the length of major axis was measured in mTmG-control and mTmG-cKO group during the time lapse imaging. n = 25 rotation events for each group. (C) Net changes of the angles of cell-cell adhesions during the chondrocyte rotation events. ****, *p*< 0.0001. unpaired t-test. mean±s.e.m. n = 25 rotation events for each group.


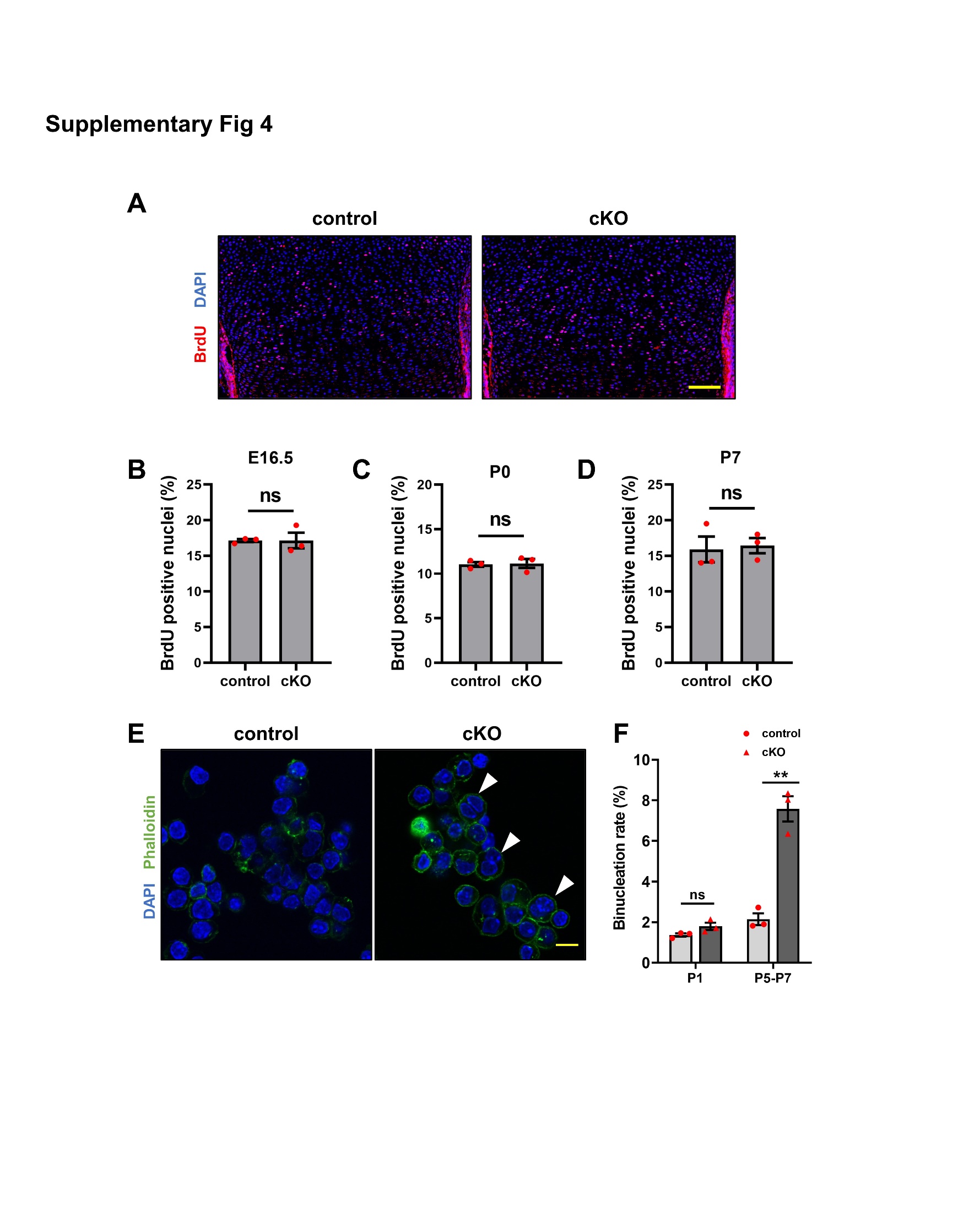


## Supplementary Figure 4. Proliferation and binucleation of cKO growth plates

(A) BrdU incorporation assays. Sections of the proximal tibia growth plates from P0 control and cKO mice were stained for BrdU (red) and counterstained with DAPI (blue). Scale bar = 100 μm. (B-D) Quantification of the ratio of BrdU positive nuclei in the proliferative zones of control and cKO growth plates at E16.5, P0, and P7. *ns*, not significant. paired t-test for P0 and P7. Unpaired t-test for E16.5. mean±s.e.m. n = 3 for each group. (E) Evaluation of binucleation. Cytosmears of control and cKO growth plate chondrocytes at P5 were stained with phalloidin (green) and DAPI (blue). White arrowheads denote the cells with more than one nucleus. Scale bar = 10 μm. (F) Quantification of the binucleation rate of P1 and P5-P7 control and cKO growth plate chondrocytes. ns, not significant. **, *p*<0.01. paired t-test. mean±s.e.m. n = 3 mice for each group. At least 200 cells were counted for binucleation for each data point.


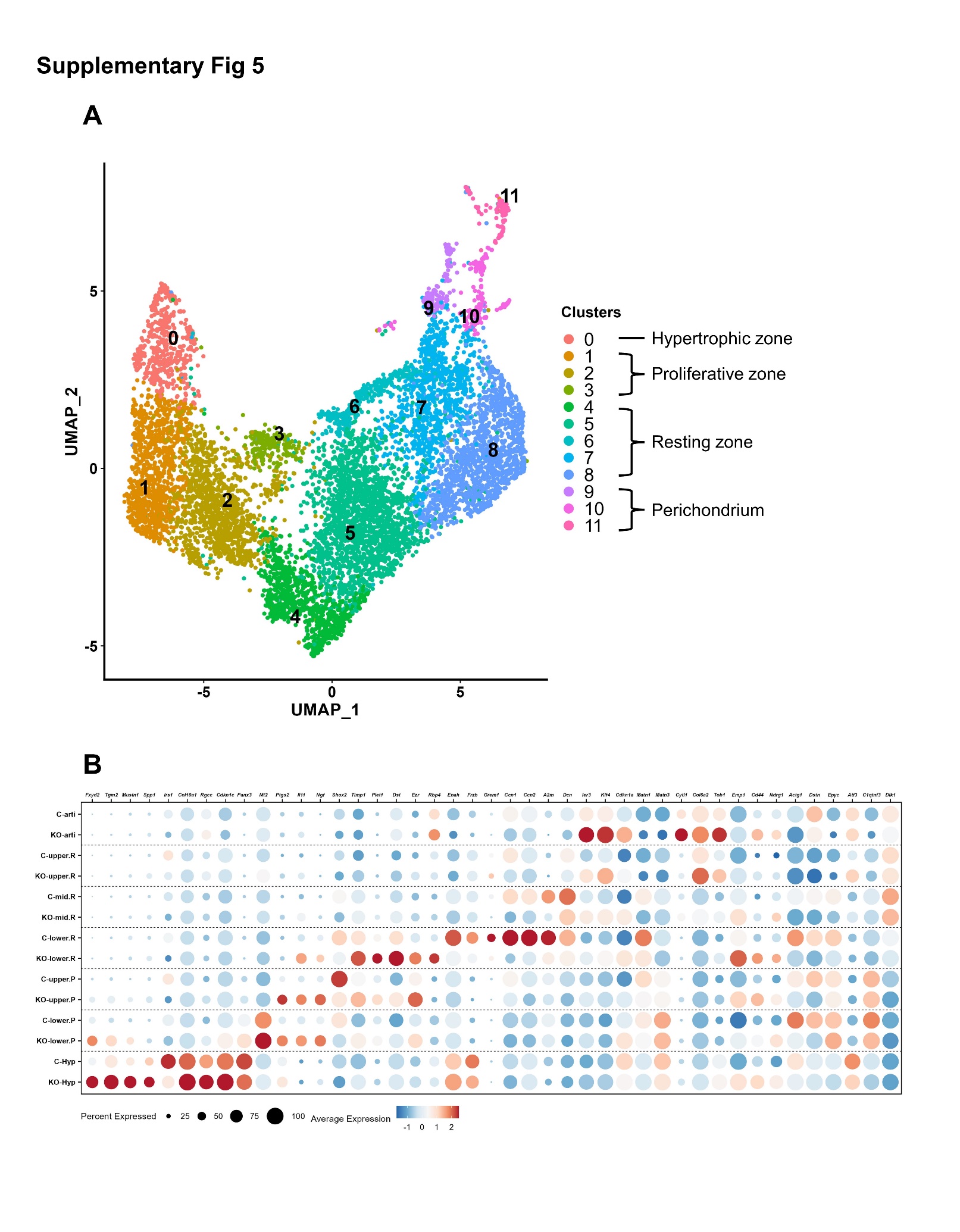


## Supplementary Figure 5. Single-cell profiling of the growth plate chondrocytes

(A) UMAP dimension reduction and clustering of the integrated data of control and cKO group. Cluster 0: hypertrophic zone. Clusters 1-3: proliferative zone. Clusters 4-8: resting zone. Clusters 9-11: perichondrium. (B) Dot plot shows the differential expression of important DEGs found in distinct zones of the growth plates.


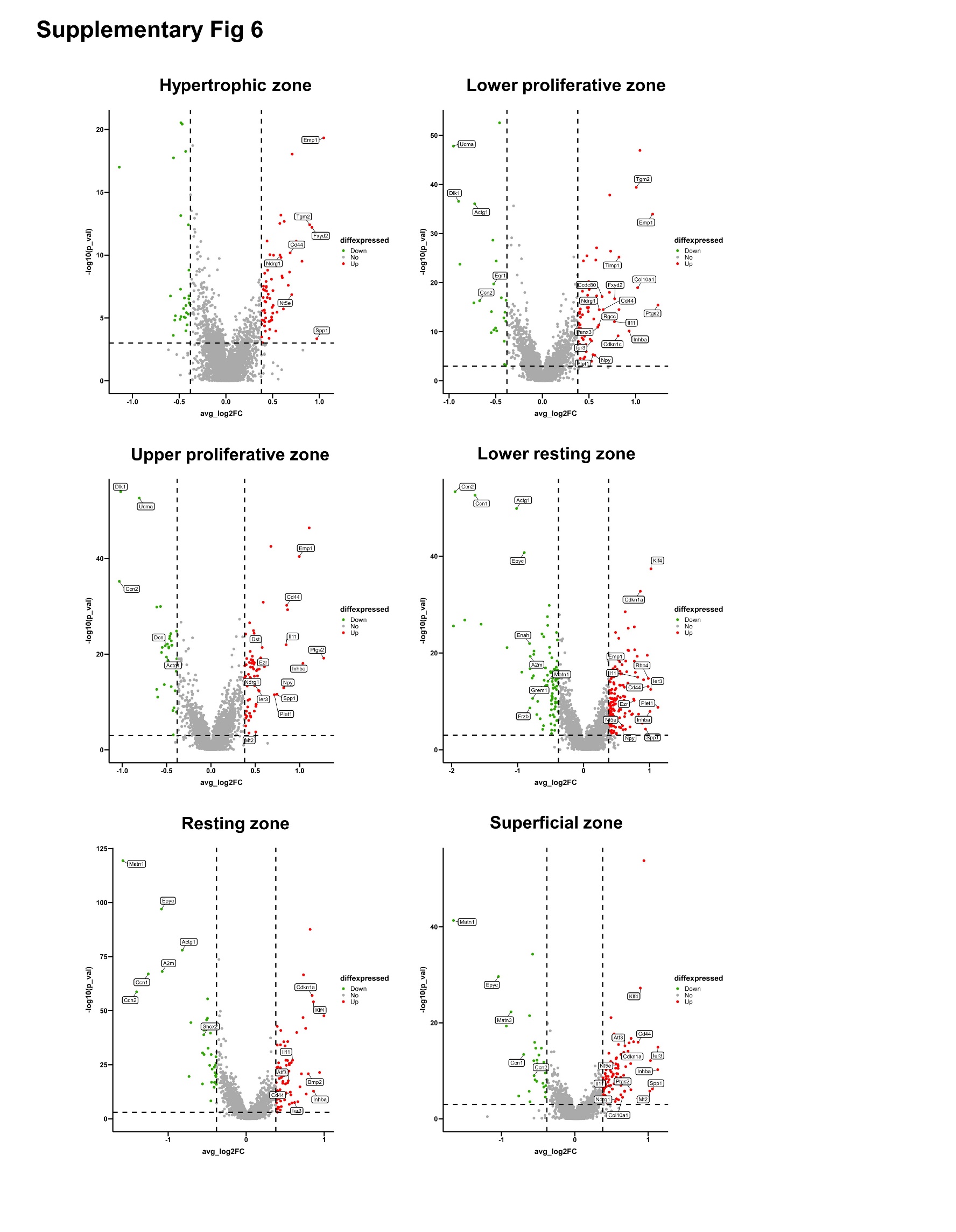


## Supplementary Figure 6. Volcano plots of genes in each zone of the growth plates

Fold changes and p values of genes in distinct zones of growth plates. Red colors denote the genes significantly upregulated in cKO chondrocytes. Green colors denote the genes significantly downregulated in cKO chondrocytes. Hypertrophic zone: Cluster 0. Lower proliferative zone: Cluster 1. Upper proliferative zone: Cluster 2. Lower resting zone: Cluster 4. Mid resting zone: Cluster 5. Superficial zone: Cluster 7. Thresholds for significant DEGs were labeled by dashed vertical lines (p < 1e-3, log2FC > 0.38, log2FC< -0.38).

##
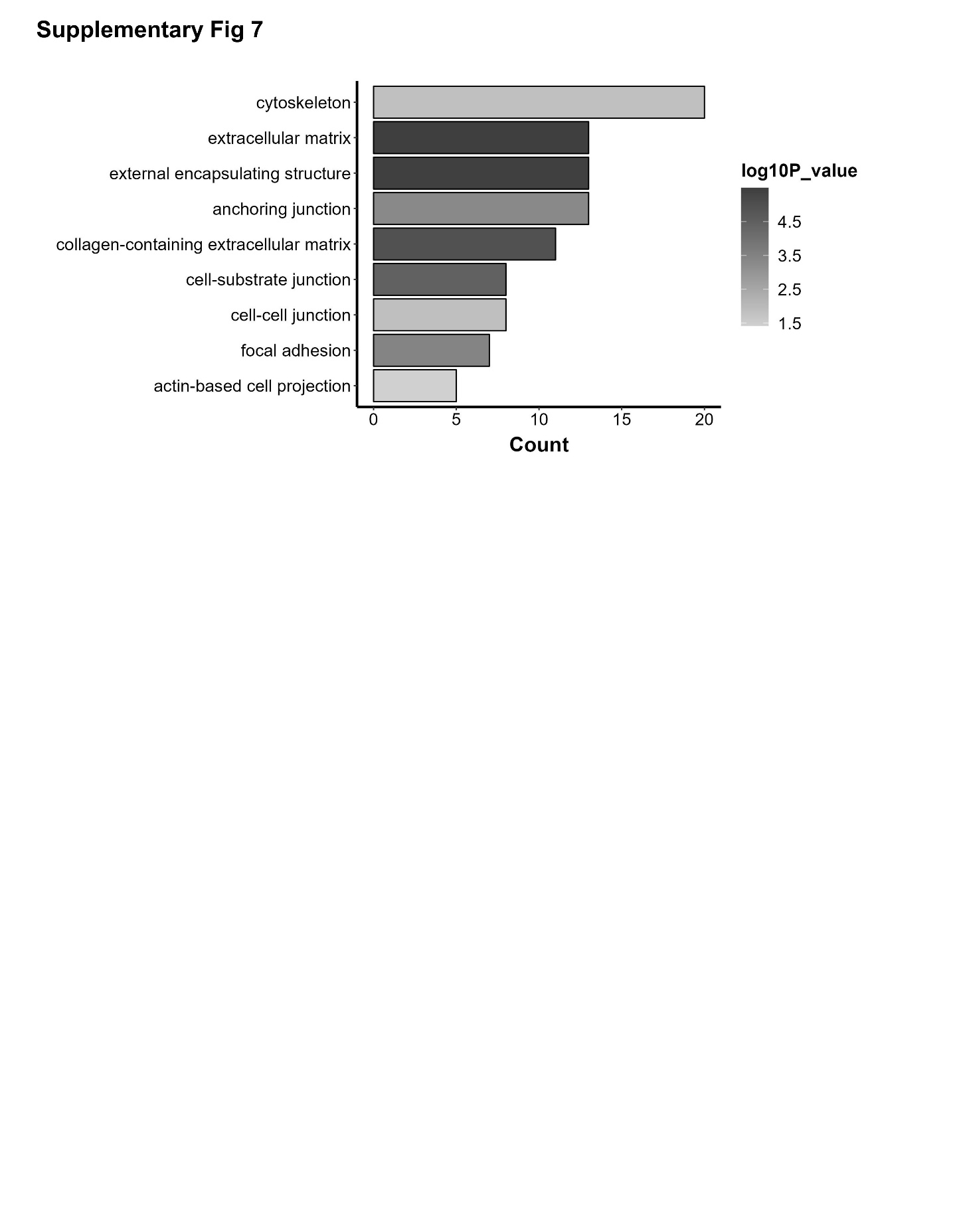
Supplementary Figure 7. Gene ontology analysis

Gene ontology analysis of DEGs of upper proliferative zone (Cluster 2). cytoskeleton, ECM, and adhesion related terms were selected and shown.

## Supplementary Movies. Two-photon time-lapse imaging of chondrocyte rotation within the growth plates of fibula explant culture.

Please refer to the folder “supplementary movies” in the .zip file of supplementary information. Movies S1-S4 show mTmG-control chondrocytes undergoing normal rotation. Movie S5 and Movie S6 show mTmG-control chondrocytes undergoing incomplete and failed rotation respectively. Movies S7 and S8 show mTmG-cKO chondrocytes undergoing normal rotation. Movies S9-S12 show mTmG-cKO chondrocytes undergoing incomplete rotation. Movies S13-S16 show mTmG-cKO chondrocytes failed in rotation. Movies S17 and S18 show mTmG-cKO chondrocytes that form aggregates during the live imaging experiments. The red arrows denote the rotating chondrocytes.

**Supplementary Table 1**

**Top 50 DEGs in the hypertrophic zone**

| **Gene** | **p_val** | **avg_log2FC** | **pct.1** | **pct.2** | **p_val_adj** |
| --- | --- | --- | --- | --- | --- |
| Rps5 | 2.9257E-21 | -0.48097163 | 1 | 1 | 9.5866E-17 |
| Rplp1 | 3.7939E-21 | -0.467855 | 1 | 1 | 1.2431E-16 |
| Emp1 | 4.7031E-20 | 1.04726758 | 1 | 1 | 1.5411E-15 |
| Rpl41 | 1.9331E-19 | -0.35544611 | 1 | 1 | 6.334E-15 |
| ENSMUSG00002075486 | 5.6521E-19 | -0.43063604 | 0.997 | 0.993 | 1.852E-14 |
| Cmss1 | 9.1518E-19 | 0.70918121 | 0.994 | 1 | 2.9988E-14 |
| Rps19 | 1.8271E-18 | -0.56044492 | 0.984 | 0.993 | 5.9869E-14 |
| Rn7sk | 9.9683E-18 | -1.14069958 | 0.53 | 0.828 | 3.2663E-13 |
| Gm15710 | 1.573E-15 | -0.37862236 | 0.575 | 0.821 | 5.1543E-11 |
| Rps25 | 3.0141E-14 | -0.36913352 | 1 | 1 | 9.8762E-10 |
| Rplp0 | 5.5168E-14 | -0.31269421 | 1 | 1 | 1.8077E-09 |
| Smox | 6.5463E-14 | 0.58855744 | 0.914 | 0.768 | 2.145E-09 |
| Ubb | 7.1807E-14 | -0.48272542 | 0.997 | 1 | 2.3529E-09 |
| Eef2 | 1.1532E-13 | -0.33535419 | 0.997 | 1 | 3.7788E-09 |
| Stxbp6 | 2.0823E-13 | 0.62425245 | 0.762 | 0.543 | 6.823E-09 |
| Col6a3 | 3.0218E-13 | 0.57704977 | 1 | 0.993 | 9.9014E-09 |
| Tgm2 | 3.8827E-13 | 0.89681453 | 0.863 | 0.748 | 1.2722E-08 |
| Ucma | 3.8938E-13 | -0.40117463 | 0.771 | 0.927 | 1.2759E-08 |
| Fxyd2 | 6.2387E-13 | 0.92062853 | 0.721 | 0.464 | 2.0442E-08 |
| Parva | 8.8249E-13 | -0.33582762 | 0.356 | 0.689 | 2.8917E-08 |
| Rps29 | 1.505E-12 | -0.32998308 | 1 | 1 | 4.9315E-08 |
| Rpl13 | 3.2612E-12 | -0.34386643 | 1 | 1 | 1.0686E-07 |
| Ptp4a2 | 7.6046E-12 | 0.440584 | 1 | 1 | 2.4918E-07 |
| Slc2a1 | 7.868E-12 | 0.75302227 | 0.962 | 0.947 | 2.5781E-07 |
| Rps28 | 8.171E-12 | -0.26834422 | 1 | 1 | 2.6774E-07 |
| Rpl23a | 2.2207E-11 | -0.30589392 | 0.997 | 1 | 7.2766E-07 |
| Rps20 | 5.6845E-11 | -0.29262944 | 1 | 1 | 1.8626E-06 |
| Cd44 | 6.6871E-11 | 0.68761389 | 0.832 | 0.636 | 2.1912E-06 |
| Rps8 | 7.3019E-11 | -0.30967684 | 1 | 1 | 2.3926E-06 |
| Il6st | 9.2403E-11 | 0.47105767 | 0.949 | 0.841 | 3.0278E-06 |
| Ndrg1 | 9.9764E-11 | 0.57883748 | 0.822 | 0.682 | 3.269E-06 |
| Sec14l1 | 1.0234E-10 | 0.50988938 | 0.94 | 0.921 | 3.3534E-06 |
| Rps21 | 1.2593E-10 | -0.27271246 | 1 | 1 | 4.1265E-06 |
| Dab2 | 1.5429E-10 | 0.58980851 | 0.87 | 0.675 | 5.0555E-06 |
| Phpt1 | 2.09E-10 | -0.30268973 | 0.546 | 0.808 | 6.8482E-06 |
| Mustn1 | 3.0642E-10 | 0.81440212 | 0.73 | 0.483 | 1.004E-05 |
| Rpl6 | 1.3026E-09 | -0.28430392 | 0.997 | 1 | 4.2683E-05 |
| Mdk | 1.4997E-09 | -0.25023411 | 0.133 | 0.384 | 4.9142E-05 |
| Fgfrl1 | 1.5697E-09 | -0.39583405 | 0.771 | 0.881 | 5.1434E-05 |
| Btg1 | 1.6139E-09 | 0.44677927 | 0.99 | 0.954 | 5.2881E-05 |
| Canx | 1.9224E-09 | -0.31010947 | 1 | 1 | 6.2992E-05 |
| Rpl22 | 2.0855E-09 | -0.28270117 | 0.997 | 1 | 6.8337E-05 |
| Pfkfb3 | 2.2086E-09 | 0.68118958 | 0.8 | 0.702 | 7.2369E-05 |
| Clic4 | 2.7573E-09 | 0.41553191 | 1 | 0.993 | 9.0349E-05 |
| Eef1b2 | 3.7868E-09 | -0.31605893 | 0.99 | 1 | 0.00012408 |
| Vldlr | 4.5988E-09 | 0.60050897 | 0.857 | 0.735 | 0.00015069 |
| Col5a2 | 6.269E-09 | 0.60442515 | 0.959 | 0.894 | 0.00020542 |
| Tmem64 | 8.175E-09 | -0.36485928 | 0.565 | 0.781 | 0.00026787 |
| Tsc22d1 | 8.2558E-09 | 0.48770622 | 0.994 | 0.987 | 0.00027052 |
| Rbms1 | 9.3834E-09 | 0.3582918 | 0.825 | 0.689 | 0.00030747 |

Top 50 DEGs in the lower proliferative zone (cluster 1)

| **Gene** | **p_val** | **avg_log2FC** | **pct.1** | **pct.2** | **p_val_adj** |
| --- | --- | --- | --- | --- | --- |
| Rps19 | 2.5644E-53 | -0.45918357 | 0.998 | 1 | 8.4027E-49 |
| Ucma | 1.504E-48 | -0.95336462 | 0.924 | 0.993 | 4.9282E-44 |
| Anxa8 | 1.1184E-47 | 1.0463368 | 0.942 | 0.794 | 3.6647E-43 |
| Tgm2 | 3.8838E-40 | 1.00699624 | 0.661 | 0.305 | 1.2726E-35 |
| Cmss1 | 1.3702E-38 | 0.72255416 | 0.996 | 0.998 | 4.4898E-34 |
| Dlk1 | 2.7543E-37 | -0.89838019 | 0.962 | 0.995 | 9.0251E-33 |
| Actg1 | 8.3156E-37 | -0.72631031 | 1 | 1 | 2.7248E-32 |
| Rps5 | 2.2161E-36 | -0.30881619 | 1 | 1 | 7.2615E-32 |
| Emp1 | 1.0886E-34 | 1.18263828 | 0.991 | 0.993 | 3.5669E-30 |
| Parva | 7.3041E-30 | -0.32888662 | 0.228 | 0.577 | 2.3933E-25 |
| C4b | 2.2062E-29 | -0.53045596 | 0.241 | 0.589 | 7.2289E-25 |
| ENSMUSG00002075486 | 2.1639E-28 | -0.33453818 | 0.998 | 0.998 | 7.0906E-24 |
| Gm15710 | 2.5893E-28 | -0.37440714 | 0.605 | 0.887 | 8.4842E-24 |
| Clic4 | 7.9159E-28 | 0.58203283 | 0.998 | 0.991 | 2.5938E-23 |
| Lgals3 | 3.7831E-27 | 0.73182026 | 0.991 | 0.969 | 1.2396E-22 |
| Bsg | 3.3225E-26 | 0.47764608 | 1 | 1 | 1.0887E-21 |
| Gapdh | 5.7813E-26 | 0.33677485 | 1 | 1 | 1.8943E-21 |
| Timp1 | 6.1453E-26 | 0.82168271 | 0.929 | 0.832 | 2.0136E-21 |
| Smox | 2.4714E-25 | 0.57295389 | 0.835 | 0.655 | 8.0981E-21 |
| Capns1 | 3.7171E-25 | 0.44003306 | 0.915 | 0.87 | 1.218E-20 |
| Shox2 | 4.0053E-25 | -0.49490671 | 0.817 | 0.941 | 1.3124E-20 |
| Hspa1a | 1.7601E-24 | -0.88431969 | 0.79 | 0.948 | 5.7673E-20 |
| Hapln1 | 2.7543E-22 | 0.37464283 | 1 | 1 | 9.0251E-18 |
| Eef2 | 2.8811E-22 | -0.26952233 | 1 | 1 | 9.4406E-18 |
| Calm1 | 5.8921E-21 | 0.49666743 | 0.976 | 0.981 | 1.9307E-16 |
| Cnnm4 | 9.5326E-21 | 0.38987041 | 0.694 | 0.48 | 3.1235E-16 |
| Egr1 | 1.8191E-20 | -0.52213241 | 0.967 | 1 | 5.9607E-16 |
| Epha2 | 2.2161E-20 | 0.35217884 | 0.482 | 0.222 | 7.2614E-16 |
| P4ha2 | 4.8609E-20 | 0.37572788 | 0.987 | 0.957 | 1.5928E-15 |
| Col10a1 | 1.0809E-19 | 1.02173956 | 0.938 | 0.877 | 3.5416E-15 |
| Cpe | 2.4146E-19 | 0.50231173 | 0.991 | 0.986 | 7.9121E-15 |
| S100a10 | 5.6249E-19 | 0.43031848 | 0.989 | 0.983 | 1.8431E-14 |
| Ngf | 9.5995E-19 | 0.71908897 | 0.674 | 0.468 | 3.1455E-14 |
| Ugdh | 3.6307E-18 | 0.48526864 | 0.996 | 0.991 | 1.1897E-13 |
| H2bc4 | 4.8254E-18 | 0.5821488 | 0.944 | 0.851 | 1.5812E-13 |
| Ccdc80 | 6.9659E-18 | 0.64082009 | 0.848 | 0.73 | 2.2825E-13 |
| Tmbim1 | 1.0691E-17 | 0.36975602 | 0.924 | 0.842 | 3.5031E-13 |
| Dstn | 1.1844E-17 | -0.44004188 | 0.975 | 0.991 | 3.881E-13 |
| Fxyd2 | 1.941E-17 | 0.77476444 | 0.607 | 0.395 | 6.36E-13 |
| Eif1 | 2.6557E-17 | 0.25075668 | 0.996 | 1 | 8.702E-13 |
| H19 | 3.8336E-17 | -0.38914922 | 0.998 | 1 | 1.2562E-12 |
| Ccn2 | 5.0096E-17 | -0.67304807 | 0.786 | 0.957 | 1.6415E-12 |
| Thbd | 5.1705E-17 | 0.48597992 | 0.275 | 0.071 | 1.6942E-12 |
| Rabac1 | 1.0018E-16 | 0.33475742 | 0.909 | 0.825 | 3.2825E-12 |
| Hspa1b | 1.3259E-16 | -0.73482293 | 0.774 | 0.927 | 4.3446E-12 |
| Acly | 1.6075E-16 | 0.44035627 | 0.944 | 0.889 | 5.2675E-12 |
| Eid1 | 2.457E-16 | -0.31565277 | 0.935 | 0.965 | 8.0509E-12 |
| Ptgs2 | 3.7522E-16 | 1.23903303 | 0.649 | 0.43 | 1.2295E-11 |
| Grb10 | 5.9569E-16 | -0.30022358 | 0.993 | 1 | 1.9519E-11 |
| Itm2c | 9.3939E-16 | -0.36233031 | 0.893 | 0.946 | 3.0781E-11 |

Top 50 DEGs in the upper proliferative zone (cluster 2)

| **Gene** | **p_val** | **avg_log2FC** | **pct.1** | **pct.2** | **p_val_adj** |
| --- | --- | --- | --- | --- | --- |
| Dlk1 | 1.07E-54 | -1.0169887 | 0.985 | 1 | 3.5061E-50 |
| Ucma | 2.306E-53 | -0.80763922 | 0.973 | 0.998 | 7.5562E-49 |
| Anxa8 | 3.8523E-47 | 1.10764819 | 0.809 | 0.586 | 1.2623E-42 |
| Cmss1 | 2.8275E-43 | 0.67599315 | 0.998 | 0.998 | 9.265E-39 |
| Emp1 | 3.762E-41 | 0.99546734 | 0.991 | 0.992 | 1.2327E-36 |
| Ccn2 | 5.7936E-36 | -1.03291918 | 0.741 | 0.953 | 1.8984E-31 |
| Clic4 | 1.375E-31 | 0.58964528 | 0.989 | 0.992 | 4.5054E-27 |
| Cd44 | 6.0648E-31 | 0.85442876 | 0.762 | 0.524 | 1.9873E-26 |
| C4b | 1.0834E-30 | -0.56841034 | 0.497 | 0.778 | 3.55E-26 |
| Hspa1a | 1.3613E-30 | -0.61025829 | 0.676 | 0.89 | 4.4605E-26 |
| Ngf | 5.0511E-30 | 0.86461136 | 0.658 | 0.378 | 1.6551E-25 |
| Rpl10 | 4.9904E-28 | 0.3224107 | 0.997 | 1 | 1.6352E-23 |
| Parva | 2.0038E-27 | -0.33736898 | 0.259 | 0.555 | 6.5659E-23 |
| Txn1 | 2.8074E-27 | 0.43637513 | 0.989 | 0.998 | 9.1992E-23 |
| S100a10 | 1.1569E-25 | 0.47973821 | 0.979 | 0.982 | 3.7909E-21 |
| Matn1 | 1.4937E-25 | -0.38796171 | 0.998 | 1 | 4.8943E-21 |
| Denr | 4.2709E-25 | 0.48676134 | 0.759 | 0.541 | 1.3994E-20 |
| Bmp5 | 4.985E-25 | -0.45252629 | 0.417 | 0.718 | 1.6334E-20 |
| Cnnm4 | 6.1422E-25 | 0.39194891 | 0.6 | 0.349 | 2.0126E-20 |
| Islr | 8.759E-25 | -0.37262868 | 0.944 | 0.994 | 2.8701E-20 |
| Sfrp1 | 1.6632E-24 | -0.46223496 | 0.532 | 0.775 | 5.4497E-20 |
| Rps6 | 2.1119E-24 | 0.286305 | 0.998 | 1 | 6.9201E-20 |
| Gapdh | 2.4423E-24 | 0.35739351 | 0.998 | 0.998 | 8.0026E-20 |
| Fos | 5.4589E-24 | -0.46722836 | 0.767 | 0.933 | 1.7887E-19 |
| Gm15710 | 1.234E-23 | -0.30760303 | 0.526 | 0.8 | 4.0434E-19 |
| Shox2 | 1.6432E-23 | -0.44651082 | 0.909 | 0.98 | 5.3844E-19 |
| Tgm2 | 2.025E-23 | 0.49742751 | 0.45 | 0.19 | 6.6352E-19 |
| Irs1 | 7.5553E-23 | -0.49046966 | 0.412 | 0.665 | 2.4756E-18 |
| Il11 | 1.0971E-22 | 0.84751739 | 0.615 | 0.375 | 3.5949E-18 |
| Egr1 | 1.3721E-22 | -0.43882941 | 0.983 | 0.994 | 4.496E-18 |
| Rian | 1.4083E-22 | -0.44217515 | 0.864 | 0.967 | 4.6146E-18 |
| Dcn | 1.7864E-22 | -0.51436747 | 0.791 | 0.914 | 5.8534E-18 |
| Meg3 | 2.9466E-22 | -0.47421834 | 0.914 | 0.978 | 9.6553E-18 |
| Nrep | 3.959E-22 | -0.54861612 | 0.835 | 0.947 | 1.2972E-17 |
| Dst | 4.0634E-22 | 0.57783361 | 0.874 | 0.767 | 1.3314E-17 |
| Tram2 | 5.0204E-22 | -0.47491159 | 0.68 | 0.831 | 1.645E-17 |
| Col1a2 | 6.8215E-22 | -0.25188919 | 0.974 | 1 | 2.2352E-17 |
| Rps19 | 8.0206E-22 | -0.25894659 | 0.998 | 1 | 2.6281E-17 |
| Rpl28 | 8.0274E-22 | 0.25797523 | 0.998 | 1 | 2.6304E-17 |
| ENSMUSG00002075486 | 2.3513E-21 | -0.28151461 | 1 | 1 | 7.7045E-17 |
| B4galt1 | 2.5915E-21 | 0.44294115 | 0.738 | 0.524 | 8.4914E-17 |
| Junb | 4.2876E-21 | -0.55693011 | 0.641 | 0.796 | 1.4049E-16 |
| Ptpre | 2.7051E-20 | 0.4669159 | 0.458 | 0.222 | 8.8639E-16 |
| Actg1 | 3.9678E-20 | -0.501316 | 0.998 | 1 | 1.3001E-15 |
| Sgms2 | 5.9614E-20 | 0.56105151 | 0.885 | 0.829 | 1.9534E-15 |
| Ptgs2 | 6.7538E-20 | 1.27188137 | 0.583 | 0.343 | 2.213E-15 |
| Col6a3 | 8.2592E-20 | 0.40371081 | 0.98 | 0.994 | 2.7063E-15 |
| Col16a1 | 8.407E-20 | -0.33495005 | 0.986 | 0.992 | 2.7547E-15 |
| Tspan6 | 9.3165E-20 | -0.3849442 | 0.726 | 0.861 | 3.0527E-15 |
| Peg3 | 1.0217E-19 | -0.35308676 | 0.977 | 0.992 | 3.3479E-15 |

Top 50 DEGs in the lower resting zone (cluster 4)

| **Gene** | **p_val** | **avg_log2FC** | **pct.1** | **pct.2** | **p_val_adj** |
| --- | --- | --- | --- | --- | --- |
| Hspb1 | 2.6977E-26 | -1.97103599 | 0.546 | 0.852 | 8.8395E-22 |
| Ccn2 | 4.8511E-54 | -1.94707448 | 0.822 | 0.984 | 1.5896E-49 |
| Hspa1a | 1.6033E-27 | -1.79937284 | 0.655 | 0.878 | 5.2536E-23 |
| Ccn1 | 2.5493E-53 | -1.64496087 | 0.589 | 0.947 | 8.3534E-49 |
| Hspa1b | 1.1139E-26 | -1.55189738 | 0.623 | 0.862 | 3.65E-22 |
| Hsph1 | 7.7654E-22 | -1.15918528 | 0.923 | 0.972 | 2.5445E-17 |
| Actg1 | 1.4281E-50 | -1.01482485 | 0.995 | 1 | 4.6796E-46 |
| Dnaja4 | 9.0447E-17 | -0.93531743 | 0.265 | 0.513 | 2.9637E-12 |
| Epyc | 1.8422E-41 | -0.8976848 | 0.963 | 0.994 | 6.0364E-37 |
| Enah | 1.1149E-22 | -0.81845114 | 0.907 | 0.966 | 3.6531E-18 |
| Frzb | 2.3206E-09 | -0.81293521 | 0.57 | 0.702 | 7.604E-05 |
| Hsp90aa1 | 1.7399E-17 | -0.8114892 | 0.995 | 0.998 | 5.701E-13 |
| A2m | 5.0361E-20 | -0.80747194 | 0.724 | 0.89 | 1.6502E-15 |
| Enc1 | 1.487E-15 | -0.77911383 | 0.371 | 0.647 | 4.8724E-11 |
| Grem1 | 2.4585E-11 | -0.77135886 | 0.281 | 0.497 | 8.0557E-07 |
| Cav1 | 4.8798E-16 | -0.75900569 | 0.647 | 0.844 | 1.599E-11 |
| Amotl2 | 1.9301E-20 | -0.75465908 | 0.477 | 0.765 | 6.3245E-16 |
| Cd24a | 3.97E-21 | -0.75120114 | 0.711 | 0.909 | 1.3009E-16 |
| Lox | 5.275E-08 | -0.70837579 | 0.292 | 0.483 | 0.00172846 |
| Cited2 | 9.8144E-11 | -0.69440683 | 0.318 | 0.511 | 3.2159E-06 |
| Dnaja1 | 3.8183E-07 | -0.65660333 | 0.918 | 0.943 | 0.01251131 |
| Thbs1 | 1.2486E-13 | -0.63890198 | 0.989 | 1 | 4.0912E-09 |
| Noct | 1.1451E-24 | -0.62968717 | 0.724 | 0.931 | 3.7521E-20 |
| Serpine1 | 5.9195E-05 | -0.61931279 | 0.581 | 0.7 | 1 |
| Cald1 | 5.7556E-13 | -0.61529111 | 0.403 | 0.627 | 1.8859E-08 |
| Cebpd | 1.0351E-05 | -0.60623968 | 0.448 | 0.574 | 0.33916302 |
| Srpx | 4.4461E-24 | -0.60378291 | 0.812 | 0.955 | 1.4569E-19 |
| Arid5b | 4.2342E-15 | -0.59697222 | 0.273 | 0.527 | 1.3874E-10 |
| Myh9 | 1.2277E-17 | -0.57681505 | 0.785 | 0.899 | 4.0228E-13 |
| Maff | 5.7705E-14 | -0.57542185 | 0.557 | 0.793 | 1.8908E-09 |
| Dlc1 | 4.4442E-14 | -0.5532763 | 0.658 | 0.83 | 1.4562E-09 |
| Col1a2 | 3.9808E-16 | -0.5472243 | 0.992 | 0.998 | 1.3044E-11 |
| Serpinh1 | 3.1515E-28 | -0.54686806 | 0.992 | 1 | 1.0327E-23 |
| Mia | 1.9452E-26 | -0.54311846 | 0.862 | 0.988 | 6.3737E-22 |
| Matn1 | 6.9153E-15 | -0.53945783 | 0.995 | 1 | 2.2659E-10 |
| P4ha1 | 1.464E-30 | -0.52024682 | 0.971 | 0.988 | 4.7969E-26 |
| Grb10 | 6.7366E-22 | -0.51576016 | 0.942 | 0.996 | 2.2074E-17 |
| Mbnl1 | 1.0803E-20 | -0.51092722 | 0.902 | 0.988 | 3.5399E-16 |
| Hmox1 | 7.7503E-08 | -0.5057914 | 0.493 | 0.653 | 0.00253953 |
| Prss35 | 6.9197E-17 | -0.50152815 | 0.488 | 0.746 | 2.2674E-12 |
| Fhl2 | 1.2115E-16 | -0.49567505 | 0.326 | 0.59 | 3.9696E-12 |
| Csf1 | 4.1655E-06 | -0.49206665 | 0.332 | 0.479 | 0.13649144 |
| Scd2 | 6.4522E-11 | -0.48895537 | 0.796 | 0.933 | 2.1142E-06 |
| Man2a1 | 5.3124E-09 | -0.48738441 | 0.305 | 0.485 | 0.00017407 |
| Cryab | 0.00043112 | -0.48506149 | 0.403 | 0.529 | 1 |
| Vim | 3.5599E-10 | -0.48498729 | 0.82 | 0.923 | 1.1665E-05 |
| Lmcd1 | 8.6742E-05 | -0.48388307 | 0.271 | 0.396 | 1 |
| Ahnak | 7.557E-12 | -0.48169064 | 0.942 | 0.986 | 2.4762E-07 |
| Tram2 | 9.4785E-20 | -0.4814058 | 0.528 | 0.797 | 3.1058E-15 |
| Cavin1 | 7.8353E-17 | -0.47558076 | 0.926 | 0.974 | 2.5674E-12 |

Top 50 DEGs in the mid resting zone (cluster 5)

| **Gene** | **p_val** | **avg_log2FC** | **pct.1** | **pct.2** | **p_val_adj** |
| --- | --- | --- | --- | --- | --- |
| Matn1 | 4.346E-120 | -1.58070964 | 0.858 | 0.992 | 1.424E-115 |
| Epyc | 9.1872E-98 | -1.08600393 | 0.748 | 0.953 | 3.0104E-93 |
| Cmss1 | 2.4853E-88 | 0.8195634 | 1 | 0.997 | 8.1434E-84 |
| Actg1 | 9.9438E-79 | -0.8199301 | 0.99 | 1 | 3.2583E-74 |
| Col9a1 | 2.0236E-74 | -0.34970385 | 1 | 1 | 6.6307E-70 |
| A2m | 7.569E-69 | -1.07706312 | 0.476 | 0.809 | 2.4801E-64 |
| Ccn1 | 1.0358E-67 | -1.25535255 | 0.686 | 0.916 | 3.3939E-63 |
| Col6a2 | 2.5558E-67 | 0.73394401 | 0.996 | 0.994 | 8.3745E-63 |
| Ccn2 | 1.8673E-59 | -1.40432514 | 0.809 | 0.95 | 6.1186E-55 |
| Cdkn1a | 9.3839E-58 | 0.84549687 | 0.942 | 0.859 | 3.0748E-53 |
| Mia | 3.3766E-56 | -0.49467443 | 0.896 | 0.985 | 1.1064E-51 |
| Klf4 | 7.6208E-55 | 0.86243418 | 0.954 | 0.906 | 2.4971E-50 |
| Col11a1 | 1.7738E-50 | -0.33076281 | 1 | 1 | 5.8121E-46 |
| Anxa8 | 2.3496E-48 | 0.99665013 | 0.617 | 0.361 | 7.6989E-44 |
| Acan | 2.5055E-48 | -0.3418503 | 1 | 1 | 8.2099E-44 |
| Sgms2 | 1.4676E-47 | 0.72898727 | 0.911 | 0.808 | 4.8089E-43 |
| Me1 | 3.2656E-47 | -0.49650238 | 0.822 | 0.947 | 1.07E-42 |
| Map3k1 | 1.7142E-46 | -0.50770886 | 0.307 | 0.626 | 5.6171E-42 |
| Tgfbi | 3.1173E-45 | -0.70837917 | 0.79 | 0.934 | 1.0215E-40 |
| Parva | 3.7505E-44 | -0.37903762 | 0.24 | 0.553 | 1.2289E-39 |
| Rpl10 | 1.8424E-43 | 0.39828833 | 0.998 | 0.999 | 6.0369E-39 |
| Sh3kbp1 | 1.5053E-42 | 0.76246375 | 0.744 | 0.559 | 4.9325E-38 |
| Rplp1 | 1.6599E-42 | -0.31173346 | 1 | 1 | 5.4389E-38 |
| Loxl2 | 6.3626E-42 | -0.51403563 | 0.637 | 0.853 | 2.0848E-37 |
| Scd2 | 9.5655E-42 | -0.5418254 | 0.811 | 0.963 | 3.1343E-37 |
| Eif5 | 1.3755E-41 | 0.44341558 | 0.992 | 0.989 | 4.507E-37 |
| Tob1 | 1.197E-40 | 0.64778812 | 0.701 | 0.524 | 3.9223E-36 |
| Prune2 | 2.2768E-40 | -0.45760922 | 0.121 | 0.378 | 7.4605E-36 |
| Shox2 | 1.38E-39 | -0.54419248 | 0.597 | 0.845 | 4.5218E-35 |
| Gnas | 3.9883E-38 | 0.31212963 | 1 | 1 | 1.3068E-33 |
| Cnmd | 1.1434E-37 | -0.28768298 | 0.998 | 1 | 3.7464E-33 |
| P4ha1 | 2.2488E-37 | -0.350041 | 0.971 | 0.998 | 7.3685E-33 |
| Col9a2 | 9.0732E-37 | -0.26295342 | 1 | 1 | 2.973E-32 |
| Mfge8 | 1.9172E-36 | 0.53264518 | 0.988 | 0.981 | 6.2821E-32 |
| Prnp | 2.3169E-36 | 0.48207485 | 0.954 | 0.939 | 7.5918E-32 |
| Fkbp9 | 3.6587E-35 | -0.32162136 | 0.988 | 0.999 | 1.1988E-30 |
| Capg | 6.1786E-35 | 0.4398678 | 0.397 | 0.166 | 2.0245E-30 |
| Col6a1 | 7.85E-35 | 0.39828362 | 1 | 1 | 2.5722E-30 |
| Serpinh1 | 1.4948E-34 | -0.32245959 | 0.994 | 1 | 4.8979E-30 |
| Pcolce | 1.9517E-34 | 0.49902562 | 0.915 | 0.869 | 6.395E-30 |
| Cavin1 | 2.4573E-33 | -0.48831183 | 0.86 | 0.951 | 8.052E-29 |
| Ssr3 | 8.9856E-32 | 0.31910531 | 0.993 | 0.995 | 2.9443E-27 |
| Fabp7 | 1.0387E-31 | -0.37913854 | 0.199 | 0.441 | 3.4036E-27 |
| Mdk | 1.6829E-31 | -0.34633011 | 0.229 | 0.474 | 5.5143E-27 |
| Junb | 3.2096E-31 | -0.55901269 | 0.606 | 0.81 | 1.0517E-26 |
| Tspan7 | 1.3622E-30 | -0.33054218 | 0.228 | 0.472 | 4.4636E-26 |
| Met | 1.912E-30 | -0.54042084 | 0.371 | 0.609 | 6.2651E-26 |
| Tram2 | 1.9536E-30 | -0.44909644 | 0.652 | 0.846 | 6.4015E-26 |
| Mif | 5.326E-30 | -0.33792398 | 0.713 | 0.899 | 1.7452E-25 |
| Egln1 | 2.0886E-29 | -0.38754355 | 0.66 | 0.832 | 6.8438E-25 |

Top 50 DEGs in the upper resting zone (cluster 7)

| **Gene** | **p_val** | **avg_log2FC** | **pct.1** | **pct.2** | **p_val_adj** |
| --- | --- | --- | --- | --- | --- |
| Cmss1 | 1.646E-54 | 0.94212793 | 0.995 | 0.994 | 5.3936E-50 |
| Matn1 | 4.546E-42 | -1.65419542 | 0.579 | 0.882 | 1.4896E-37 |
| Parva | 4.8657E-35 | -0.57482863 | 0.17 | 0.558 | 1.5943E-30 |
| Epyc | 2.2456E-30 | -1.04076941 | 0.463 | 0.782 | 7.3582E-26 |
| Klf4 | 5.6114E-28 | 0.89284285 | 0.966 | 0.879 | 1.8387E-23 |
| Matn3 | 5.1552E-23 | -0.87022937 | 0.749 | 0.903 | 1.6892E-18 |
| Col9a1 | 3.2119E-22 | -0.61720018 | 0.995 | 1 | 1.0524E-17 |
| Hspa5 | 8.1288E-22 | 0.49342323 | 0.998 | 1 | 2.6636E-17 |
| Rplp1 | 1.2713E-20 | -0.37079208 | 1 | 1 | 4.1656E-16 |
| Rn7sk | 4.568E-20 | -0.93350909 | 0.692 | 0.869 | 1.4968E-15 |
| Rps29 | 4.5316E-19 | -0.33472622 | 1 | 1 | 1.4849E-14 |
| Col6a2 | 2.0274E-18 | 0.53561282 | 1 | 0.997 | 6.6433E-14 |
| Nfkbia | 1.7698E-17 | 0.76747621 | 0.757 | 0.57 | 5.7992E-13 |
| Csrnp1 | 8.8462E-17 | 0.80124751 | 0.599 | 0.352 | 2.8986E-12 |
| Cd44 | 1.0928E-16 | 0.86241137 | 0.535 | 0.29 | 3.5807E-12 |
| Mia | 1.172E-16 | -0.54721134 | 0.692 | 0.863 | 3.8402E-12 |
| Ppp1r15a | 1.5157E-16 | 0.74029538 | 0.803 | 0.642 | 4.9663E-12 |
| Tob1 | 3.2936E-16 | 0.59590071 | 0.827 | 0.651 | 1.0792E-11 |
| Atf3 | 6.275E-16 | 0.68347514 | 0.778 | 0.57 | 2.0561E-11 |
| Ier3 | 1.2024E-15 | 1.13307215 | 0.821 | 0.738 | 3.9399E-11 |
| Cnmd | 1.9117E-15 | -0.45585421 | 0.956 | 0.991 | 6.2642E-11 |
| Col9a2 | 1.9677E-15 | -0.53237265 | 0.989 | 1 | 6.4476E-11 |
| Cdkn1a | 8.0754E-15 | 0.72590726 | 0.868 | 0.791 | 2.6461E-10 |
| Rps25 | 1.0302E-14 | -0.2835226 | 1 | 1 | 3.3756E-10 |
| Pou3f3 | 1.1742E-14 | -0.31888363 | 0.098 | 0.293 | 3.8476E-10 |
| Rbbp6 | 1.399E-14 | 0.66774775 | 0.847 | 0.695 | 4.5842E-10 |
| Eif4a1 | 2.2082E-14 | 0.46443005 | 0.912 | 0.838 | 7.2357E-10 |
| Anxa8 | 3.8527E-14 | 0.62863557 | 0.649 | 0.452 | 1.2624E-09 |
| Ccn1 | 3.9641E-14 | -0.69675368 | 0.79 | 0.913 | 1.2989E-09 |
| Snorc | 5.0073E-14 | -0.48970926 | 0.824 | 0.928 | 1.6407E-09 |
| Ier5 | 1.878E-13 | 0.56916728 | 0.765 | 0.583 | 6.1536E-09 |
| Nfkbiz | 3.2524E-13 | 0.56160097 | 0.649 | 0.439 | 1.0657E-08 |
| Irf2bpl | 6.1892E-13 | -0.51475986 | 0.378 | 0.611 | 2.028E-08 |
| Cxcl1 | 7.4839E-13 | 1.03106447 | 0.38 | 0.156 | 2.4522E-08 |
| Scd2 | 7.8033E-13 | -0.55911255 | 0.693 | 0.832 | 2.5569E-08 |
| Rnf19b | 1.1259E-12 | 0.48954858 | 0.527 | 0.321 | 3.6892E-08 |
| X7SK | 1.5319E-12 | -0.42233214 | 0.282 | 0.514 | 5.0195E-08 |
| Pnp | 1.7532E-12 | 0.49824213 | 0.586 | 0.383 | 5.7448E-08 |
| Has1 | 2.7555E-12 | 0.51398099 | 0.312 | 0.106 | 9.0289E-08 |
| Hbegf | 3.0934E-12 | 0.76157603 | 0.778 | 0.626 | 1.0136E-07 |
| Col6a1 | 3.2818E-12 | 0.39531805 | 0.998 | 0.997 | 1.0754E-07 |
| Rn18s.rs5 | 3.2967E-12 | 0.39118945 | 1 | 1 | 1.0802E-07 |
| Sfrp1 | 3.434E-12 | -0.51279 | 0.3 | 0.498 | 1.1252E-07 |
| S100a10 | 3.5343E-12 | 0.55967268 | 0.899 | 0.81 | 1.1581E-07 |
| Brd2 | 4.1985E-12 | 0.48084129 | 0.869 | 0.779 | 1.3757E-07 |
| Nr4a2 | 4.9526E-12 | 0.58705717 | 0.84 | 0.723 | 1.6228E-07 |
| Ddx3x | 6.3634E-12 | 0.43336228 | 0.959 | 0.935 | 2.0851E-07 |
| Rps19 | 7.1237E-12 | -0.32120771 | 0.993 | 0.988 | 2.3342E-07 |
| Nt5e | 9.8617E-12 | 0.59019496 | 0.773 | 0.617 | 3.2314E-07 |
| Arf4 | 1.0044E-11 | 0.27604461 | 0.989 | 0.981 | 3.2912E-07 |

Top 50 DEGs in the articular chondrocytes (cluster 8)

| **Gene** | **p_val** | **avg_log2FC** | **pct.1** | **pct.2** | **p_val_adj** |
| --- | --- | --- | --- | --- | --- |
| Cmss1 | 9.3067E-67 | 0.99194184 | 0.996 | 0.985 | 3.0495E-62 |
| Matn1 | 5.5844E-66 | -1.87132728 | 0.536 | 0.911 | 1.8298E-61 |
| Epyc | 1.5837E-53 | -1.32841234 | 0.395 | 0.798 | 5.1893E-49 |
| Matn3 | 1.7086E-47 | -1.2192374 | 0.588 | 0.881 | 5.5985E-43 |
| C1qtnf3 | 3.1556E-45 | -1.10370062 | 0.451 | 0.81 | 1.034E-40 |
| Col9a1 | 2.2123E-42 | -0.82886585 | 1 | 1 | 7.2491E-38 |
| A2m | 4.1066E-42 | -0.7635311 | 0.095 | 0.418 | 1.3456E-37 |
| Mia | 2.819E-38 | -0.74549657 | 0.483 | 0.795 | 9.237E-34 |
| Klf4 | 2.897E-34 | 0.78806761 | 0.994 | 0.958 | 9.4925E-30 |
| Rn18s.rs5 | 1.0981E-31 | 0.61921464 | 1 | 1 | 3.5981E-27 |
| Rplp1 | 5.2785E-30 | -0.39705311 | 1 | 1 | 1.7296E-25 |
| Cnmd | 4.4798E-28 | -0.59676982 | 0.959 | 0.994 | 1.4679E-23 |
| Actg1 | 2.1836E-26 | -0.68130386 | 0.978 | 1 | 7.155E-22 |
| Parva | 3.7356E-25 | -0.39089732 | 0.168 | 0.457 | 1.2241E-20 |
| Cxcl14 | 1.0287E-24 | -0.50817062 | 0.188 | 0.484 | 3.3708E-20 |
| Ucma | 2.0112E-24 | -0.78879111 | 0.877 | 0.955 | 6.5901E-20 |
| Rps29 | 2.3068E-24 | -0.33109382 | 0.999 | 1 | 7.5588E-20 |
| Colgalt2 | 3.7398E-24 | -0.38898412 | 0.148 | 0.415 | 1.2254E-19 |
| Dcn | 1.0426E-23 | -0.76796473 | 0.585 | 0.819 | 3.4162E-19 |
| Col6a2 | 1.5501E-23 | 0.45538988 | 1 | 1 | 5.0791E-19 |
| Slc38a2 | 5.5724E-23 | 0.58641782 | 0.966 | 0.932 | 1.8259E-18 |
| Rn7sk | 6.0282E-23 | -1.03455353 | 0.408 | 0.688 | 1.9753E-18 |
| Tubb2b | 1.8791E-22 | -0.33296977 | 0.09 | 0.312 | 6.1573E-18 |
| Mfge8 | 2.9214E-22 | 0.59013702 | 0.989 | 0.988 | 9.5727E-18 |
| Sfrp1 | 3.9946E-22 | -0.46147709 | 0.177 | 0.442 | 1.3089E-17 |
| Nt5e | 4.54E-22 | 0.79869071 | 0.879 | 0.81 | 1.4876E-17 |
| Cytl1 | 5.2833E-22 | 1.12179709 | 0.763 | 0.549 | 1.7312E-17 |
| Snorc | 1.1308E-21 | -0.55867793 | 0.782 | 0.914 | 3.7052E-17 |
| Clic4 | 2.2451E-21 | 0.51984384 | 0.992 | 0.982 | 7.3567E-17 |
| Hspa1a | 4.9106E-21 | -1.20942796 | 0.751 | 0.917 | 1.6091E-16 |
| Enah | 8.8973E-21 | -0.63713853 | 0.472 | 0.724 | 2.9154E-16 |
| Smoc2 | 9.6484E-21 | -0.53943812 | 0.388 | 0.668 | 3.1615E-16 |
| Hspb1 | 1.5792E-20 | -1.18916364 | 0.556 | 0.801 | 5.1747E-16 |
| Chadl | 5.1295E-20 | -0.48425389 | 0.281 | 0.534 | 1.6808E-15 |
| Tob1 | 7.9674E-20 | 0.60340043 | 0.872 | 0.774 | 2.6107E-15 |
| Gm15710 | 1.089E-19 | -0.3126345 | 0.233 | 0.522 | 3.5684E-15 |
| Col9a2 | 1.1114E-19 | -0.50200507 | 0.996 | 1 | 3.6418E-15 |
| Cd9 | 1.7115E-19 | 0.39979555 | 0.994 | 0.985 | 5.608E-15 |
| Rpl41 | 3.0679E-19 | -0.28006652 | 1 | 1 | 1.0052E-14 |
| Fabp7 | 6.9932E-19 | -0.41053263 | 0.077 | 0.258 | 2.2915E-14 |
| Ccn2 | 2.7979E-18 | -0.76923933 | 0.851 | 0.967 | 9.1679E-14 |
| Scube3 | 3.4433E-18 | -0.41541377 | 0.228 | 0.475 | 1.1283E-13 |
| Mir6236 | 7.0948E-18 | 0.69149609 | 0.545 | 0.386 | 2.3248E-13 |
| Ccn1 | 1.0357E-16 | -0.78415096 | 0.777 | 0.929 | 3.3937E-12 |
| Ier3 | 1.3825E-16 | 0.85967508 | 0.918 | 0.852 | 4.5301E-12 |
| Lum | 1.687E-16 | -0.4642122 | 0.481 | 0.73 | 5.5276E-12 |
| Rps5 | 2.6872E-16 | -0.27093195 | 0.996 | 0.997 | 8.8053E-12 |
| Prdx1 | 4.5246E-16 | 0.6302604 | 0.918 | 0.911 | 1.4826E-11 |
| Pou3f3 | 4.5567E-16 | -0.28632052 | 0.073 | 0.237 | 1.4931E-11 |
| Btg1 | 8.469E-16 | 0.53393403 | 0.903 | 0.869 | 2.775E-11 |

Supplementary Table 1. Differential gene expression analysis

Lists of DEGs in distinct zones of growth plates. pct.1, percentage of cells expressing the gene in cKO group. pct.2, percentage of cells expressing the gene in the control group. avg_log2FC, fold change in the cKO group compared to the control group. negative values indicate lower expression in cKO group, positive values indicate that the genes are upregulated in the cKO group.
